# Dengue viruses cleave STING in humans but not in nonhuman primates, their presumed natural reservoir

**DOI:** 10.7554/eLife.31919

**Published:** 2018-03-20

**Authors:** Alex C Stabell, Nicholas R Meyerson, Rebekah C Gullberg, Alison R Gilchrist, Kristofor J Webb, William M Old, Rushika Perera, Sara L Sawyer

**Affiliations:** 1 Department of Molecular, Cellular and Developmental BiologyUniversity of Colorado BoulderBoulderUnited States; 2 Arthropod-borne and Infectious Diseases Laboratory, Department of Microbiology, Immunology and PathologyColorado State UniversityFort CollinsUnited States; Harvard Medical SchoolUnited States

**Keywords:** zoonosis, viral reservoir, interferon, Virus

## Abstract

Human dengue viruses emerged from primate reservoirs, yet paradoxically dengue does not reach high titers in primate models. This presents a unique opportunity to examine the genetics of spillover versus reservoir hosts. The dengue virus 2 (DENV2) - encoded protease cleaves human STING, reducing type I interferon production and boosting viral titers in humans. We find that both human and sylvatic (reservoir) dengue viruses universally cleave human STING, but not the STING of primates implicated as reservoir species. The special ability of dengue to cleave STING is thus specific to humans and a few closely related ape species. Conversion of residues 78/79 to the human-encoded ‘RG’ renders all primate (and mouse) STINGs sensitive to viral cleavage. Dengue viruses may have evolved to increase viral titers in the dense and vast human population, while maintaining decreased titers and pathogenicity in the more rare animals that serve as their sustaining reservoir in nature.

## eLife digest

Dengue viruses are found in over 100 countries and cause the tropical disease known as dengue fever. Dengue viruses affect around 100 million people per year and can – in severe cases – lead to death. Unlike many other deadly diseases, there is currently no vaccine that completely prevents dengue fever.

It is thought that dengue viruses that circulate in human populations were derived from monkey versions of that same virus. However, research suggests that both human and primate variations of dengue viruses appear to multiply much better in humans than in other species. Scientists believe that this is because some animals, including primates, have defense mechanisms that are ineffective in humans.

To explore this idea, Stabell et al. looked at a protein called STING in humans and in three different primates: the chimpanzee, the rhesus macaque, and the common marmoset. STING plays an important role in the immune system and helps to fight infections caused by viruses and other microbes.

During replication – the process by which a virus spreads through an organism’s cells – the dengue virus cuts and inactivates the human STING protein, and so helps the virus spread. Stabell et al. discovered that in most primates, dengue viruses cannot inactivate STING. This was found to be reliant on a small region in the STING protein that differed between humans and primates. This small difference may, in part, explain why dengue viruses replicate better in humans than other primates.

Stabell et al. then searched for other animals whose STING protein would be susceptible to dengue virus inactivation. Using a database with genetic information of over 5,000 mammals, Stabell et al. identified STING proteins of three types of apes and three types of rodents that could also be deactivated by dengue viruses.

To develop a vaccine or antiviral drug scientists generally need to study the disease in living animals. Since dengue viruses replicate more successfully in humans than they do in other animal models, it makes it more challenging to find an effective treatment. The results from Stabell et al. may help to identify animals that could be strong candidates for future research into dengue viruses, potentially paving the way for further therapeutic development.

## Introduction

**Figure 1.**
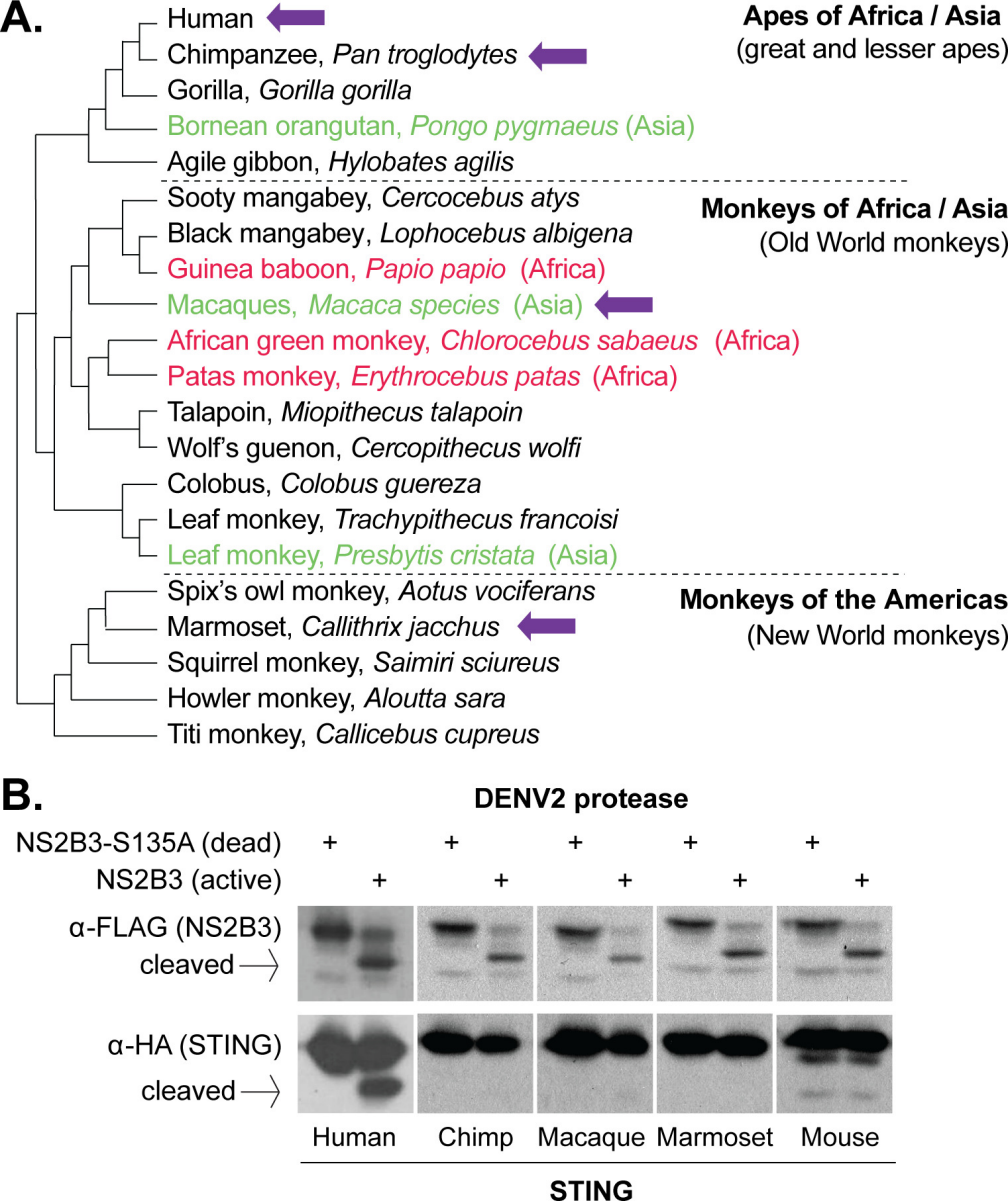
Dengue virus (DENV2) can cleave human but not nonhuman primate STING. (**A**) A phylogeny of select primate species, showing the three main simian clades: apes, Old World monkeys, and New World monkeys (Perelman et al., 2011). The primate species from which STING is tested in this study are shown with purple arrows. Possible primate reservoir hosts for sylvatic dengue viruses, based on virus isolation from sentinel monkeys, or antibody detection, are shown in red (Africa) and green (Asia). The current evidence for these primate reservoir hosts is reviewed in the discussion section. (**B**) 293T cells were cotransfected with plasmids encoding STING-HA, and the NS2B3-Flag protease complex with or without the S135 inactivating mutation. Whole cell lysate isolated 24 hr post transfection was run on a protein gel and immunoblotted with anti-Flag or anti-HA antibodies. The encoded NS2B-NS3-Flag polyprotein auto-processes into the NS2B3 protease complex if the protease is active, as seen in the anti-Flag blot where in some samples the NS3-Flag protein has been liberated through cleavage. We sometimes see lower bands underneath the full-length mouse STING, but conclude that they are endogenous degradation products since they are equal in intensity in the presence of the active or dead protease.

Dengue viruses cause clinical disease in approximately 100 million individuals each year and are found in over 100 countries (Bhatt et al., 2013). Yet, to date no vaccine exists that conveys cross-protection against all human dengue viruses (Scherwitzl et al., 2017). Dengue viruses are positive sense RNA viruses in the family *Flaviviridae,* and are related to yellow fever virus, Zika virus, and West Nile virus (Best, 2016). These viruses are primarily transmitted between humans in highly populated areas by *Aedes aegypti* and *Aedes albopictus* mosquitoes, in what are referred to as human (or ‘urban’) transmission cycles (Diamond and Pierson, 2015; Hanley et al., 2013; Vasilakis et al., 2011). Sylvatic (i.e. forest) dengue virus transmission cycles, which are separate from the human transmission cycles, exist in Asia and Africa and involve nonhuman primates and forest-dwelling *Aedes* mosquitos (Vasilakis et al., 2011; Wang et al., 2000; Rico-Hesse, 1990). While the exact nonhuman primate species that serve as the sustaining natural reservoirs for sylvatic dengue viruses are unknown, the global distribution of both dengue viruses and their transmitting mosquitoes could be consistent with a significant number of primate species being involved (Figure 1—figure supplement 1) (Hanley et al., 2013; Vasilakis et al., 2011). Primarily, dengue viruses have been associated with monkeys (rather than apes) found in Africa and Asia (Figure 1). Human dengue viruses cluster into four phylogenetically distinct clades referred to as DENV1, 2, 3, and 4 (Vasilakis and Weaver, 2008). These clades have sylvatic dengue virus isolates at their bases, supporting zoonotic origins of the four dengue viruses that now circulate in humans (Wang et al., 2000; Pyke et al., 2016; Weaver and Vasilakis, 2009). Human dengue viruses have now become uncoupled from the sylvatic reservoir and require only humans and mosquitoes to be sustained (Mayer et al., 2017).

**Figure 1—figure supplement 1.**
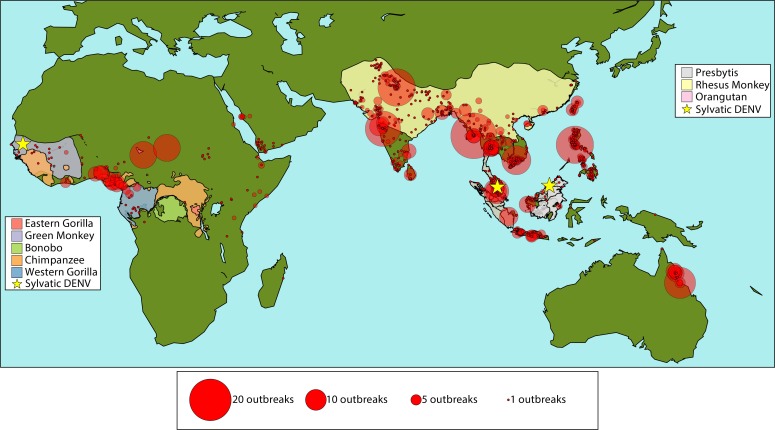
Many primate species reside in areas where dengue viruses are endemic in humans.

In side-by-side experiments, sylvatic and human dengue viruses replicate similarly in human cells (Vasilakis et al., 2007; Vasilakis et al., 2008). These results have been interpreted to mean that there is little or no adaptive barrier for the emergence of sylvatic dengue viruses into human populations, and the view that dengue viruses are generalists capable of infecting a wide range of primate species including humans. Thus, a paradox exists in understanding why human dengue viruses are so difficult to model in nonhuman primates. Chimpanzees (*Pan troglodytes*) (Scherer et al., 1978), rhesus macaques (*Macaca mulatta*) (Halstead et al., 1973; Hickey et al., 2013), marmosets (multiple *Callithrix* species) (Moi et al., 2013; Ferreira et al., 2014), and other nonhuman primate species (Althouse et al., 2014) have been explored as possible primate models for studying dengue virus pathogenesis and for vaccine challenge. In general, it has been observed that dengue does not replicate to high titers in these models, and little or no overt disease pathology is observed (Cassetti et al., 2010; Zompi and Harris, 2012). If human and sylvatic viruses are the same in their properties, we speculated that there must instead be something special about the replication of these viruses in the human host.

STING is a multi-pass transmembrane protein found in the endoplasmic reticulum, and functions as a critical component in the innate immune sensing pathway for intracellular pathogens (Ishikawa and Barber, 2008; Zhong et al., 2008; Ishikawa et al., 2009; Jin et al., 2008; Sun et al., 2009; Burdette and Vance, 2013). Although originally described as part of the response to cytosolic DNA sensing (Zhang et al., 2011), STING is also activated upon RNA virus infection (Holm et al., 2016). Underscoring this, several RNA viruses encode proteins that antagonize or degrade STING (Sun et al., 2012; Nitta et al., 2013; Ding et al., 2013; Aguirre et al., 2012; Yu et al., 2012). For instance, the NS2B3 protease of one human dengue virus, DENV2, has been shown to target human STING for cleavage (Aguirre et al., 2012; Yu et al., 2012). Through the cleavage of STING, DENV2 renders the host unable to induce the phosphorylation of Interferon Regulatory Factor 3 (IRF3), therefore decreasing production of type I interferon and increasing viral titers (Green et al., 2014). Mouse STING is resistant to cleavage by the DENV2 protease (Aguirre et al., 2012; Yu et al., 2012). This at least partially explains why mice mount an effective immune response against dengue viruses, protecting them against infection and compromising their utility as model organisms (Cassetti et al., 2010; Zompi and Harris, 2012; Ashour et al., 2010). Dengue viruses are known to mute the host interferon response in other ways as well, with the other predominant mechanism being the degradation of STAT2 (Ashour et al., 2009; Jones et al., 2005; Mazzon et al., 2009; Best, 2017; Morrison et al., 2012).

In this study, we show that the NS2B3 proteases of human (DENV1-4) and sylvatic dengue viruses universally cleave human STING. However, none of these proteases can cleave the STING proteins of chimpanzees, macaques, or marmosets, three primate species that have been pursued as model organisms. We show that an ‘RG’ motif at positions 78/79 of STING is critical for susceptibility to cleavage, and conversion of these residues to ‘RG’ renders all nonhuman primate STING proteins tested, as well as mouse STING, sensitive to dengue virus proteases. Out of the entire Genbank database, along with our sequencing of STING from 16 additional primate species, we identify only a small number of apes (gorillas, orangutans, and gibbons), and three small rodent species (chinchillas, naked mole rats, and desert woodrats) as encoding a functional dengue virus cleavage determinant in STING. This may, in part, explain why modeling dengue virus in animal models has been so difficult.

## Results

### The protease of human dengue virus, DENV2, cleaves only human STING

To begin, we cloned STING from chimpanzee (*Pan troglodytes*, Genbank XM_016953921), rhesus macaque (*Macaca mulatta*, Genbank MF622060), and the common marmoset (*Callithrix jacchus*, Genbank MF622061). These species have been explored as animal models of dengue infection, and also represent the three major clades of simian primates: apes (represented by chimpanzee), Old World monkeys (represented by macaque), and New World monkeys (represented by marmoset; Figure 1A). Most suspected dengue virus reservoir hosts belong to the Old World monkey clade (red and green type in Figure 1A). On the other hand, New World monkeys (such as marmosets), which reside exclusively in the Americas, have presumably never been exposed to sylvatic dengue viruses since sylvatic cycles do not exist in the New World. We also included human (Genbank MF622062) and mouse (*Mus musculus*, Genbank MF622063) STING in our studies as positive and negative controls, since it was previously shown that human but not mouse STING is sensitive to DENV2 NS2B3 cleavage (Aguirre et al., 2012; Yu et al., 2012).

The dengue virus NS2B3 protease complex is composed of the viral non-structural proteins NS2B and NS3 (Preugschat et al., 1990; Zhang et al., 1992; Falgout et al., 1991). In the dengue virus genome, the NS2B and NS3 genes sit adjacent and are cotranslated as part of a single long viral polyprotein (Perera and Kuhn, 2008; Chambers et al., 1990). When the NS2B - NS3 region is expressed from a plasmid, the region is translated into a small polyprotein that then auto-cleaves itself to become the functional protease complex (Yusof et al., 2000; Bera et al., 2007). We used a plasmid expressing the NS2B-NS3 region, including a 3x Flag tag at the C-terminus of NS3, from the New Guinea C isolate of DENV2 (see methods). As a control, a mutation was created at the active-site serine, changing it to an alanine (S135A), which renders the protease inactive (Rodriguez-Madoz et al., 2010). We then used a previously established cotransfection assay (Aguirre et al., 2012; Yu et al., 2012) to determine if the dengue virus protease could cleave primate STING orthologs. Plasmids encoding primate or mouse STING, and either active or S135A (dead) NS2B3 dengue proteases, were cotransfected into 293T cells. STING cleavage was assessed 24 hr later by western blot. The inactivity of the S135A protease can be seen in the anti-Flag blot, where the NS2B-NS3 polyprotein does not self-cleave when this mutation is present (Figure 1B). We see only a fraction of the human STING being cleaved, but this is consistent with previous publications and is presumably exacerbated by the overexpression of STING achieved in transfection experiments (Aguirre et al., 2012; Yu et al., 2012). Unexpectedly, none of the nonhuman primate STINGs tested were susceptible to cleavage (Figure 1B). Remarkably, the DENV2 protease could not even cleave chimpanzee STING, which differs from human STING at only three amino acid positions.

### Mapping the dengue virus cleavage determinants in STING

**Figure 2.**
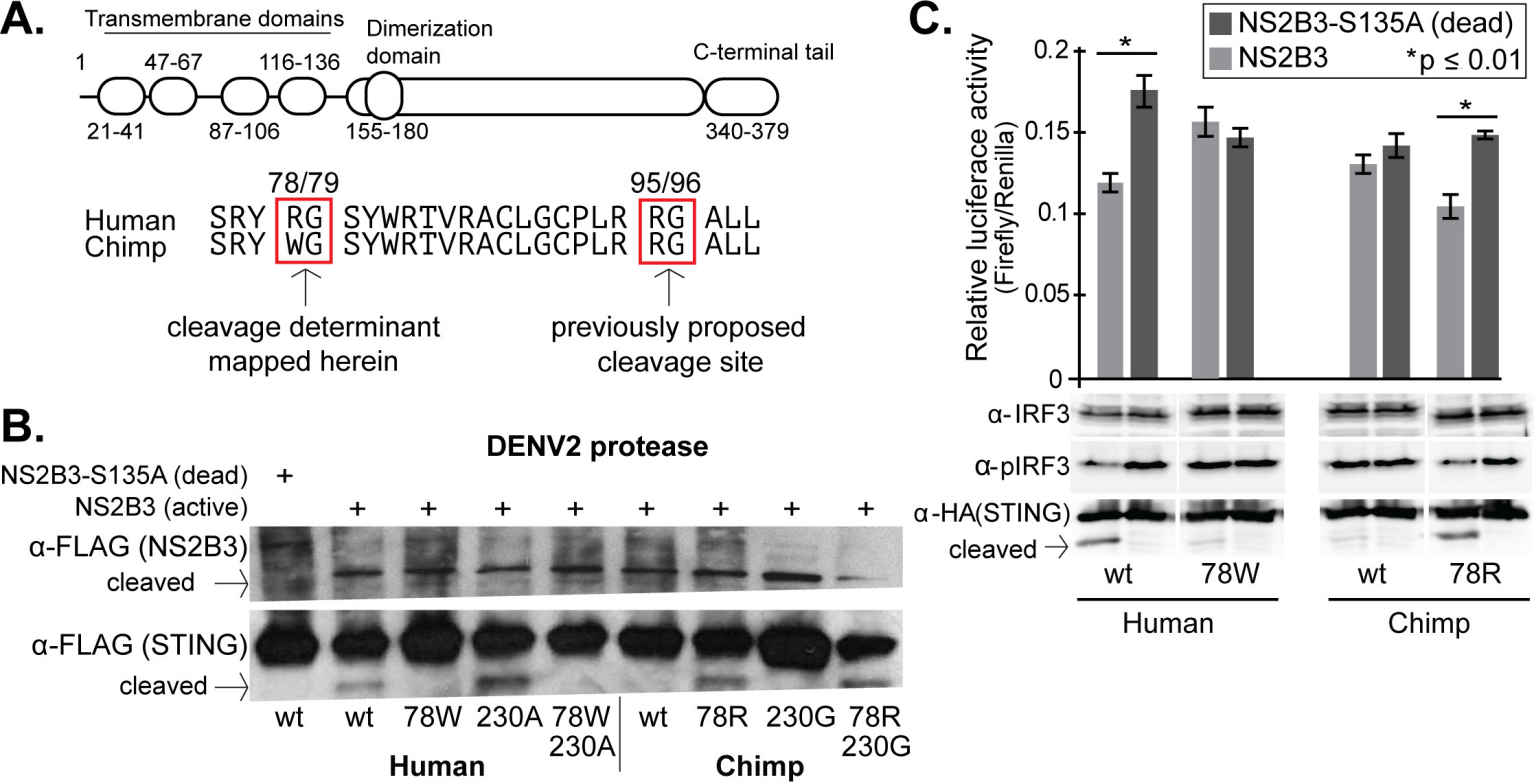
STING residue 78 determines susceptibility to NS2B3 cleavage in human versus chimpanzee STING comparisons. (**A**) A domain diagram of human STING is shown, as defined in (Wu et al., 2014). An alignment of human and chimpanzee STING in the region of the newly identified cleavage determinant (78/79) and the one previously determined (95/96) (Aguirre et al., 2012; Yu et al., 2012). (**B**) Site-directed mutagenesis was performed on either human or chimpanzee STING at position 78, substituting the residue at this position in human (R) with that in chimpanzee (W) and vice versa. Plasmids encoding the NSB3 protease complex and STING were cotransfected into 293T cells, and 48 hr later lysates were collected and analyzed by anti-FLAG western blot. In this experiment, both the protease and STING are tagged with FLAG. Data presented are representative of at least two experiments. (**C**) (bottom) 293T cells were transfected with plasmids expressing the DENV2 NS2B3 protease and wildtype (wt) or mutated (78W or 78R) STING. IRF3 and phosphorylated IRF3 (pIRF3) were detected by western blot in lysates harvested 48 hr later. (top) The identical experiment, but performed in biological triplicate and with the addition of plasmids encoding a firefly luciferase gene driven by the interferon beta (IFNb) promoter, and a renilla luciferase gene driven by a CMV promoter. The relative luciferase activity (Y-axis) was calculated by normalizing the luciferase signal to the renilla signal in each replicate. A Welch’s T-test was used to compare the levels of luciferase produced in the presence of active versus dead protease. Data is representative of at least two experiments.

The dengue virus cleavage site in STING was previously mapped to between the 95th and 96th residues (Aguirre et al., 2012; Yu et al., 2012). Some uncertainty existed, though, because in the previous studies it was noted that the human residues around 95/96 were not sufficient to convey cleavage susceptibility to mouse STING. Indeed, human and chimpanzee STING proteins have the exact same amino acid sequence at these positions (Figure 2A). Human and chimpanzee STING differ at only three amino acid positions, residues 78, 230, and 232. We found that mutating the human STING to encode the chimpanzee residue at site 78 (78W) caused it to become resistant to cleavage by DENV NS2B3 (Figure 2B). Likewise, mutating the chimpanzee STING at residue 78 to the human amino acid (78R) rendered the chimpanzee STING susceptible to cleavage (Figure 2B). We saw no effect of mutations at a second site, 230, either alone or in combination with residue 78 (Figure 2B). Previously, it had been shown that STING site 78 may be important for retention in the endoplasmic reticulum (ER) (Sun et al., 2009). To ensure that ER retention was not disrupted by the mutations that we tested, we disrupted both copies of STING in A549 cells using CRISPR-Cas9 targeting, and then stably re-complemented them with wildtype or 78W (cleavage resistant) human STING (Figure 2—figure supplement 1). Both the wildtype and mutant STING similarly localized to the ER (Figure 2—figure supplement 2). It is logical that the 78W substitution would not affect ER-localization of STING, since 78W is naturally occurring in the chimpanzee STING protein. Therefore, we can conclude that position 78R is a critical determinant for dengue virus cleavage, either as a binding site or a cleavage site for the dengue protease. It was previously estimated that STING is cleaved in a way that divides the protein into approximately 25% and 75% of its original molecular weight, with the N-terminus of the protein representing the smaller portion (Yu et al., 2012). This would place the cleavage site in the vicinity of the 78th residue. In addition, the 78/79 ‘RG’ motif is in good agreement with what is known about the preferences of NS2B3, where glycine (G) often lies directly downstream of the peptide cleavage site, and an arginine (R) directly upstream (Li et al., 2005a).

**Figure 2—figure supplement 1.**
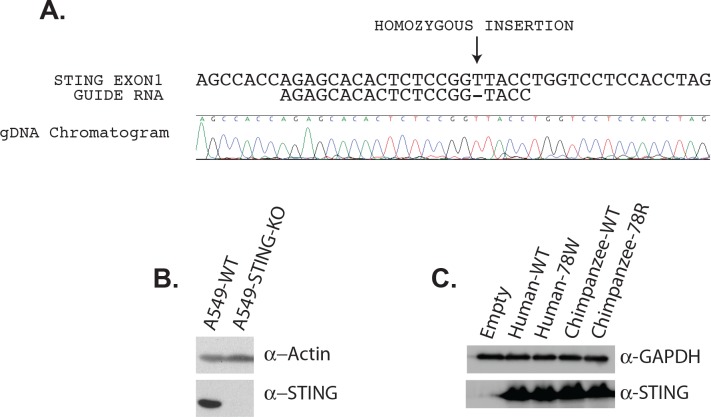
Generation of STING knockout cells using CRISPR-Cas9, and stable re-complementation of these lines. (**A**) A depiction of the insertion induced in the first exon of STING in A549 cells. Below, a Sanger sequencing chromatogram from a this genomic locus shows the homozygous insertion. (**B**) Both wildtype (wt) A549 cells and A549 cells that were knocked out for STING were probed by western blot for Actin or STING. An antibody recognizing the endogenous STING was used (anti-STING; Abcam 92605). (**C**) STING-KO A549 cells were re-complimented with HA-tagged versions of STING using lentiviral transduction. Cell lines were generated to express either wildtype (wt) or mutant STING from human or chimpanzee. A mutation was made in human STING to render it cleavage-resistant (78W). A mutation was made in chimpanzee STING to make it cleavage-susceptible (78R). Western blots where performed with antibodies against GAPDH or STING.

**Figure 2—figure supplement 2.**
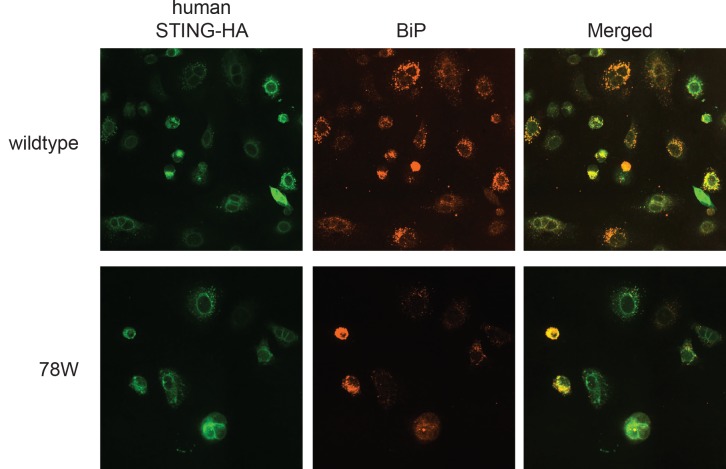
Wildtype and mutated human STING both colocalize with the ER-resident protein BiP. Immunofluorescence imaging was performed on STING and the ER-resident protein BiP. A549 cells knocked out for STING were reconstituted with either wildtype human STING (top) or human STING 78W (cleavage resistant) by retroviral transduction (as shown in Figure 2—figure supplement 1). These cells were fixed in a 4% paraformaldehyde solution. Antibodies against the HA epitope tag (STING) or BiP were incubated with the fixed cells. Cells were imaged at 40x magnification on the Nikon A1R confocal microscope.

Next we wished to ensure that the cleavage of STING alters its ability to signal in the interferon induction pathway. Transfection of plasmids encoding STING into cells is sufficient to activate the interferon induction pathway (Ishikawa and Barber, 2008). We again performed cotransfection of plasmids encoding STING and the dengue virus protease. 48 hr after transfection, cell lysates were probed in western blots for phosphorylated IRF3 (pIRF3) and for total IRF3. We found that pIRF3 was reduced when human or chimpanzee STING was susceptible to NS2B3 cleavage, and not reduced when STING was resistant to cleavage (Figure 2C, bottom). We also monitored the activation of the interferon-beta (IFNb) promoter. We performed an identical cotransfection assay with plasmids encoding STING and NS2B3, only in triplicate, and with two additional plasmids: one encoding a firefly luciferase reporter gene downstream of the IFNb promoter, and another encoding a renilla luciferase reporter gene downstream of a CMV promoter (used to normalize transfection efficiencies between samples, by taking the ratio of firefly:renilla luciferase). With human STING and the version of chimpanzee STING rendered sensitive to cleavage (78R), there was a significant reduction in firefly luciferase production in the presence of active NS2B3, in comparison to the catalytically dead version of the protease (Figure 2C, top). This reduction is not observed with chimpanzee STING, or with human STING rendered resistant to cleavage by the 78W mutation.

**Figure 3.**
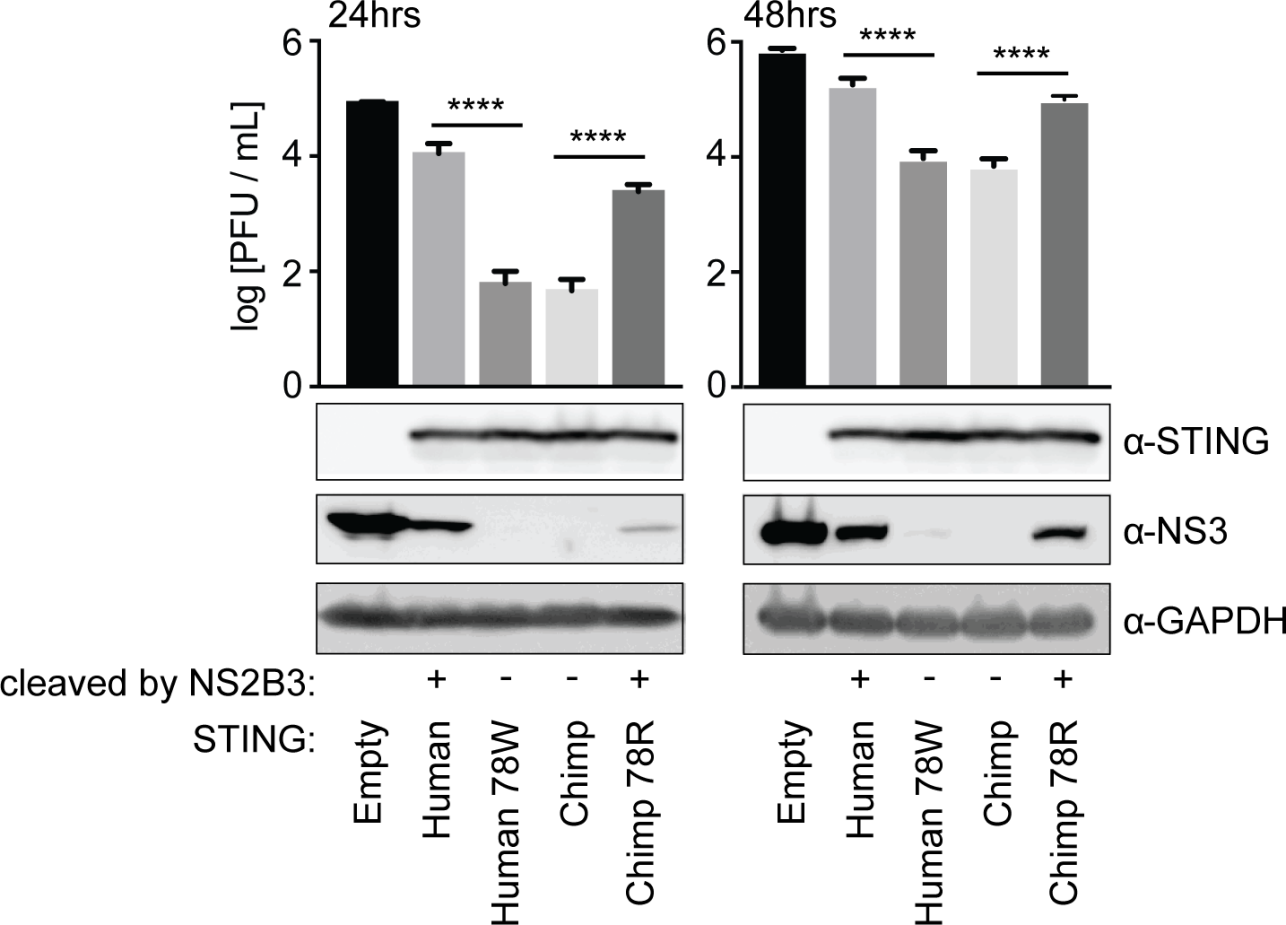
Cleavage of STING at position 78/79 promotes virus replication. The endogenous copies of STING in A549 cells were knocked out using the Cas9 nuclease (see Figure 2—figure supplement 1). These cells were re-complemented by retroviral transduction with no gene (pLPCX-empty), wildtype human STING, cleavage-resistant human STING (human 78W), wildtype chimpanzee STING, or cleavage-susceptible chimpanzee STING (chimp 78R). These cell lines were infected at MOI of 0.3 with dengue virus 2 (DENV2 16681). After 24 and 48 hr the virus supernatant was removed and titrated on BHK21 cells. At the same time, cells were collected in RIPA buffer, lysed, and run on a gel for western blotting using antibodies against STING, dengue virus NS3, and GAPDH (loading control). A Tukey's multiple comparisons test indicated significant differences in infectious virus in the presence of each mutant STING compared to wildtype STING, as shown (****=p < 0.0001), after significant one-way ANOVA. Data are representative of at least two independent experiments.

We then verified these results with infection experiments. We stably re-complemented our A549 STING knockout cells, using retroviral transduction, to express various forms of STING: chimpanzee or human 78W (both cleavage resistant), human or chimpanzee 78R (both cleavage susceptible), or cells were complemented with an empty vector (Figure 2—figure supplement 1). These cells were infected with dengue virus 2 (strain 16681) at MOI 0.3. At 24 and 48 hr post infection, supernatant was harvested and viral content was quantified by plaque assay on BHK21 cells, and at the same time cells were harvested and lysed for western blot. We found that A549 cells re-complemented with STING, regardless of the version, produced less dengue virus than the STING knockout cell line that was not re-complemented (Figure 3). However, cells re-complemented with a cleavage-resistant STING produced less virus than those re-complemented with a cleavage-susceptible STING (Figure 3). In fact, cell lines in this experiment that differ by only a single amino acid in STING demonstrate as much as a 176-fold change in infectious virus produced at 24 hr post-infection, according to the titration experiments (human versus human 78W STING). The difference remains significant at 48 hr post-infection. This suggests that cleavage of STING is critically important for dengue virus replication, and has a large impact on viral titers. The STING cleavage product was not visible in the western blots performed during these experiments. This cleavage product is typically only detectable when cells are treated with MG132 proteasome inhibitor for several hours before cell lysis. While our transfection-based cleavage assays typically incorporate MG132 treatment (see Materials and methods), it was not used in the infection experiments shown here in order to not perturb infectious virus produced. In a separate experiment performed in the presence of MG132, we do see the cleavage of STING during infections (Figure 3—figure supplement 1). Further, the cleavage of endogenous STING during dengue infection was previously demonstrated under other conditions (Aguirre et al., 2012; Yu et al., 2012).

**Figure 3—figure supplement 1.**
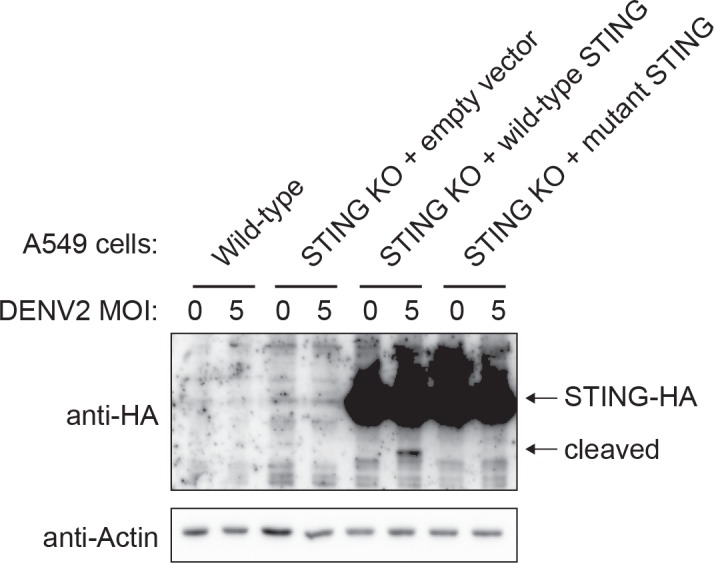
STING is cleaved during dengue virus infection. The indicated A549 cells lines (wild-type, STING knockout, or re-complemented) were plated in F-12K media with 10% FBS. Dengue virus 2 (16681) was diluted in F-12K media with 2% FBS, added to cells at an MOI of 5, and allowed to attach to cells for 1 hr at room temperature. Uninfected wells were incubated with F-12K media with 2% FBS not containing virus. F-12K media with 10% FBS was added to cells and they were maintained at 37°C with 5% CO_2_. After 20 hr the media in all wells was replaced with F-12K media with 2% FBS containing 20 μM proteasome inhibitor MG-132 (Sigma-Aldrich M7449). 4 hr following MG-132 treatment (24 hr following infection) the cells were collected and lysed in RIPA buffer supplemented with protease inhibitor (Roche, 4693159001). Protein concentration was calculated using the Bradford method. 15% 37.5:1 Acrylamide/Bisacrylamide gels were used to separate 30 μg of whole cell lysate for each sample. Protein was transferred overnight at 30 volts onto a nitrocellulose membrane. Blocking was performed with a 10% milk solution in tris-buffered saline supplemented with 0.1% TWEEN20. Primary antibodies were used against HA (3F10 clone Sigma 11867423001) and β-actin (Santa Cruz Biotechnology Sc47778).

**Figure 4.**
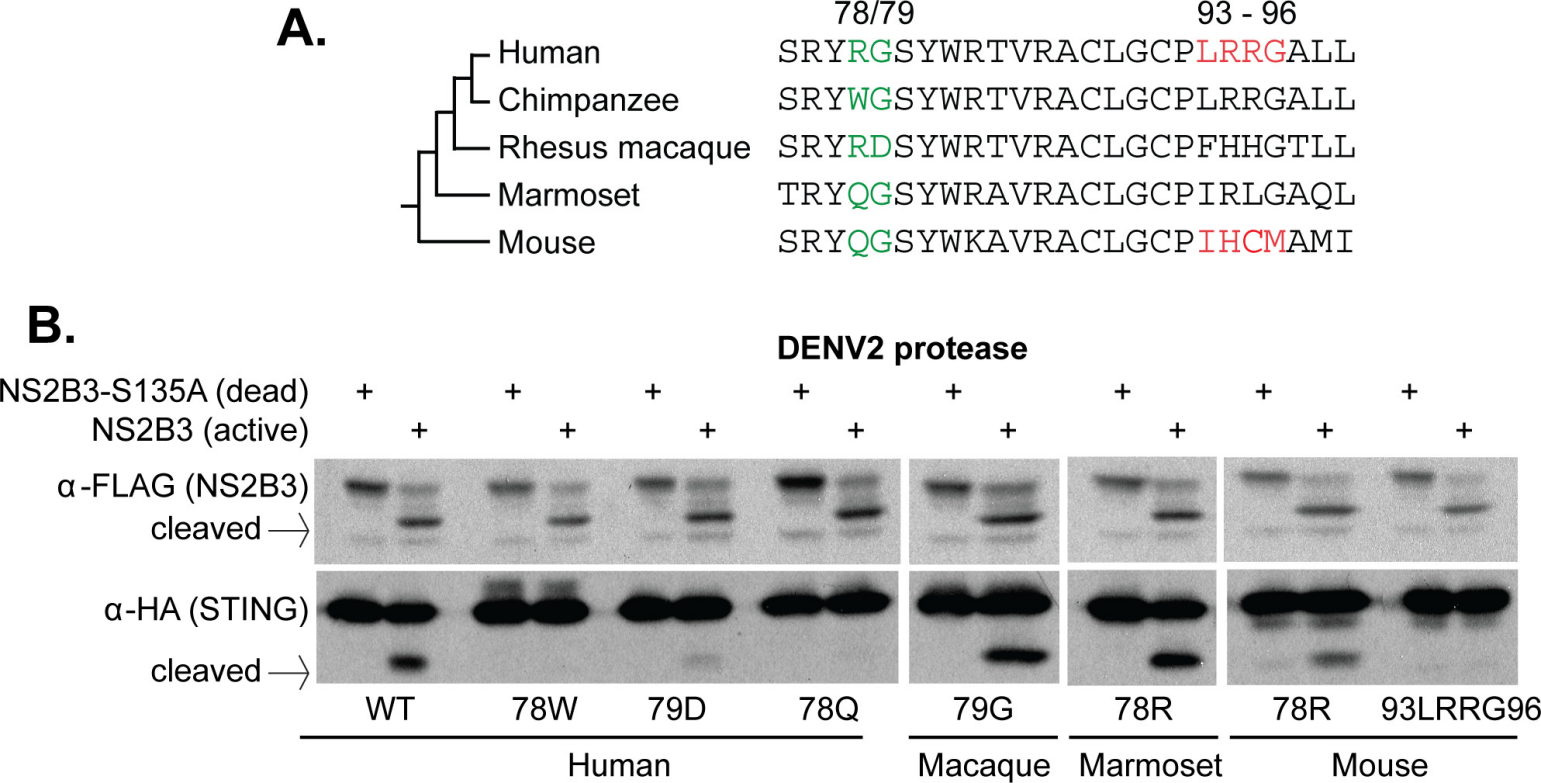
Residues 78 and 79 of STING define a dengue virus cleavage determinant in both primate and mouse STING. (**A**) A phylogeny and multiple sequence alignment of STING from various primate species and mouse. Shown in green is the 78/79 motif in STING that is mutated in panel B. Shown in red is the motif changed in mouse STING, only, in panel B. (**B**) Site directed mutagenesis was performed on human, rhesus macaque, marmoset, or mouse STING at sites 78/79 or 93–96 (mouse only). 293T cells were cotransfected with mammalian expression plasmids encoding STING along with wildtype or mutant NS2B3. 24 hr after transfection, whole-cell lysate was harvested and probed for FLAG or HA by western blot. Data presented are representative of at least two experiments.

We next wanted to determine if our newly identified cleavage determinant could explain the resistance to STING cleavage seen in other species. The dengue protease also cannot cleave rhesus macaque, marmoset, or mouse STING (Figure 1B), all of which deviate from the ‘RG’ motif found in human STING (highlighted green in Figure 4A). We next performed site-directed mutagenesis to alter either the 78th or 79th residue in STING of these species. We found that, in all cases, mutations that restored this motif to the human ‘RG’ restored susceptibility to cleavage (Figure 4B). Consistent with previous studies (Aguirre et al., 2012; Yu et al., 2012), mutation of residues 93–96 in mouse STING to match the human ‘LRRG’ did not confer susceptibility to cleavage by NS2B3 (Figure 4B). Overall, these results further support the conclusion that sites 78 and 79 are critical determinants for cleavage by the DENV NS2B3 protease. An ‘RG’ motif at these two positions is both necessary and sufficient to make primate and rodent STING susceptible to cleavage by the DENV2 protease.

### The 78–79 RG motif in STING is a universal cleavage determinant for the proteases of human and sylvatic dengue viruses

**Figure 5.**
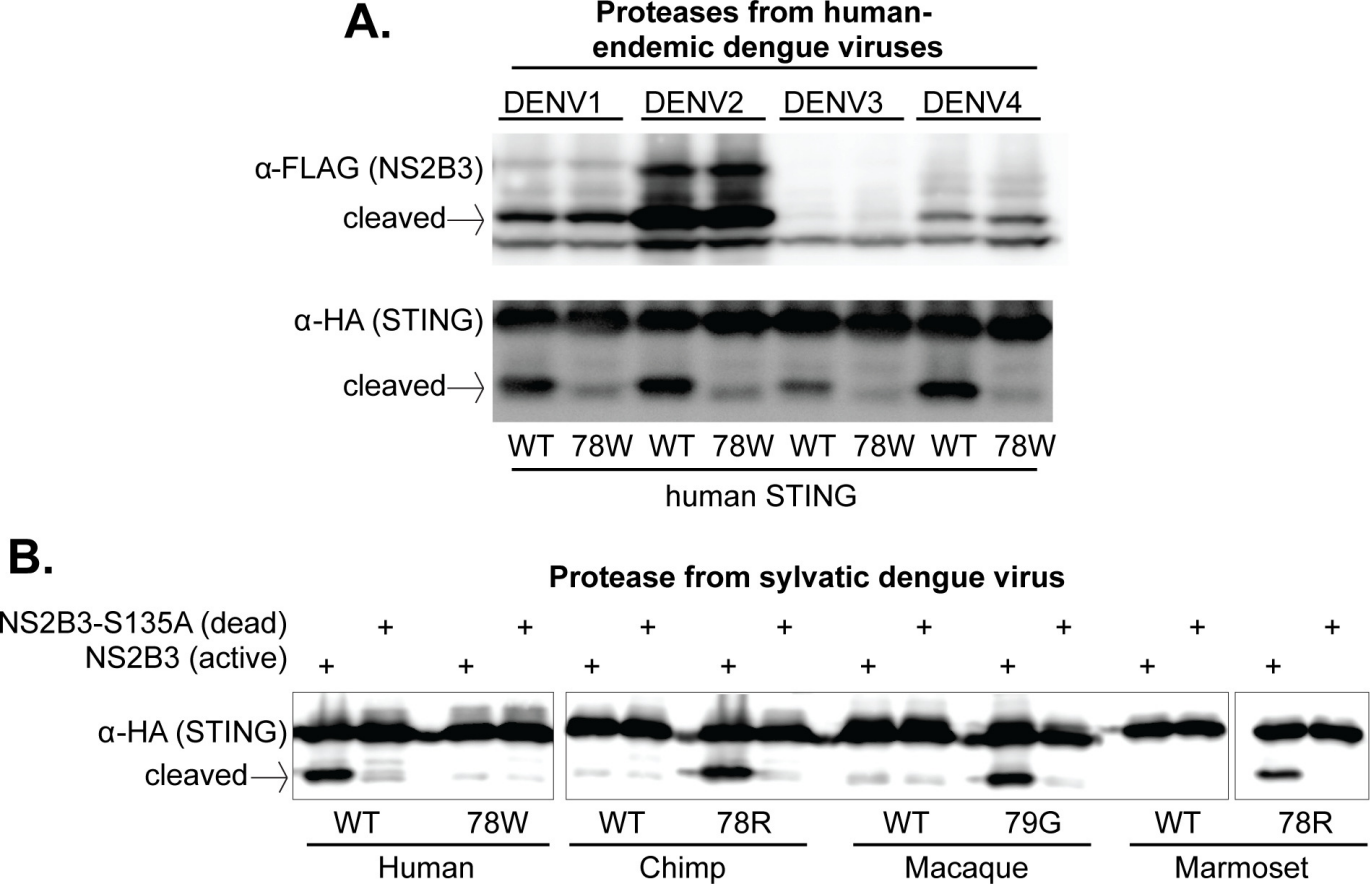
The 78/79 cleavage determinant in STING is targeted by proteases encoded by all endemic human dengue viruses, and by at least one sylvatic dengue virus. (**A**) 293T cells were cotransfected with plasmids encoding NS2B3 from DENV1-4 along with human STING with or without a mutation at site 78. Western blotting was performed on lysate harvested 24 hr post transfection to detect NS2B3 (anti-FLAG) or STING (anti-HA). Data presented are representative of at least two experiments. (**B**) 293T cells were cotransfected with plasmids encoding the indicated STING and the NS2B3 from a sylvatic isolate of dengue virus (DakAr-141069). 24 hr post transfection, lysates where harvested, run on a gel, and western blotting was performed with an anti-HA antibody to detect STING. All data presented are representative of at least two experiments.

To test whether these results are generalizable to other dengue viruses endemic in humans, we cloned the region encoding the NS2B3 protease complex from three additional DENV isolates (one from each endemic human virus): DENV1 (Hawaii), DENV3 (Philippines/H887/1956), and DENV4 (H241). While some of these proteases expressed better than others, all were able to cleave wildtype human STING far more efficiently than human STING bearing the 78W mutation (Figure 5A). This data indicates that the 78/79 RG motif of STING is recognized (i.e. bound or cleaved) by the NS2B3 proteases of all endemic human dengue viruses. If residues 78/79 in fact constitute the actual cleavage site for the protease, this would be in line with biochemical studies showing that the proteases of all four endemic human dengue viruses have similar cleavage motif preferences (Li et al., 2005a).

**Figure 5—figure supplement 1.**
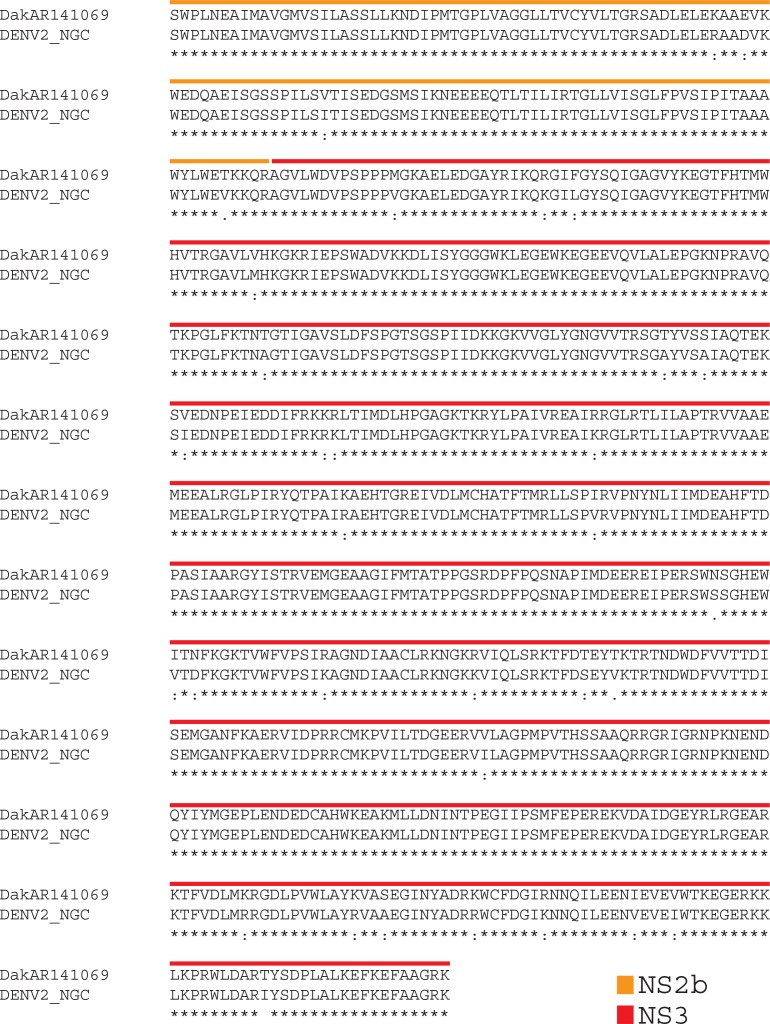
Alignment of the NS2B3 protease from sylvatic (top) versus human (bottom) dengue viruses.

We next cloned the NS2B3 protease from a sylvatic dengue strain (DakAr-141069). This virus was first isolated from an *Ae. luteocephalus* mosquito in Senegal in 1999 (Vasilakis et al., 2008). We find that this viral protease also cleaves human STING, but not the STING of chimpanzee, rhesus macaque, or marmoset (Figure 5B). Further, the restoration of the ‘RG’ motif at positions 78/79 again renders all of these STING proteins susceptible to cleavage (Figure 5B), indicating that the sylvatic protease is targeting (i.e. binding or cleaving) the same cleavage determinant as the proteases from human dengue viruses. This is consistent with the high degree of similarity between human and sylvatic proteases, as can be seen in alignment of the two (Figure 5—figure supplement 1).

**Figure 6.**
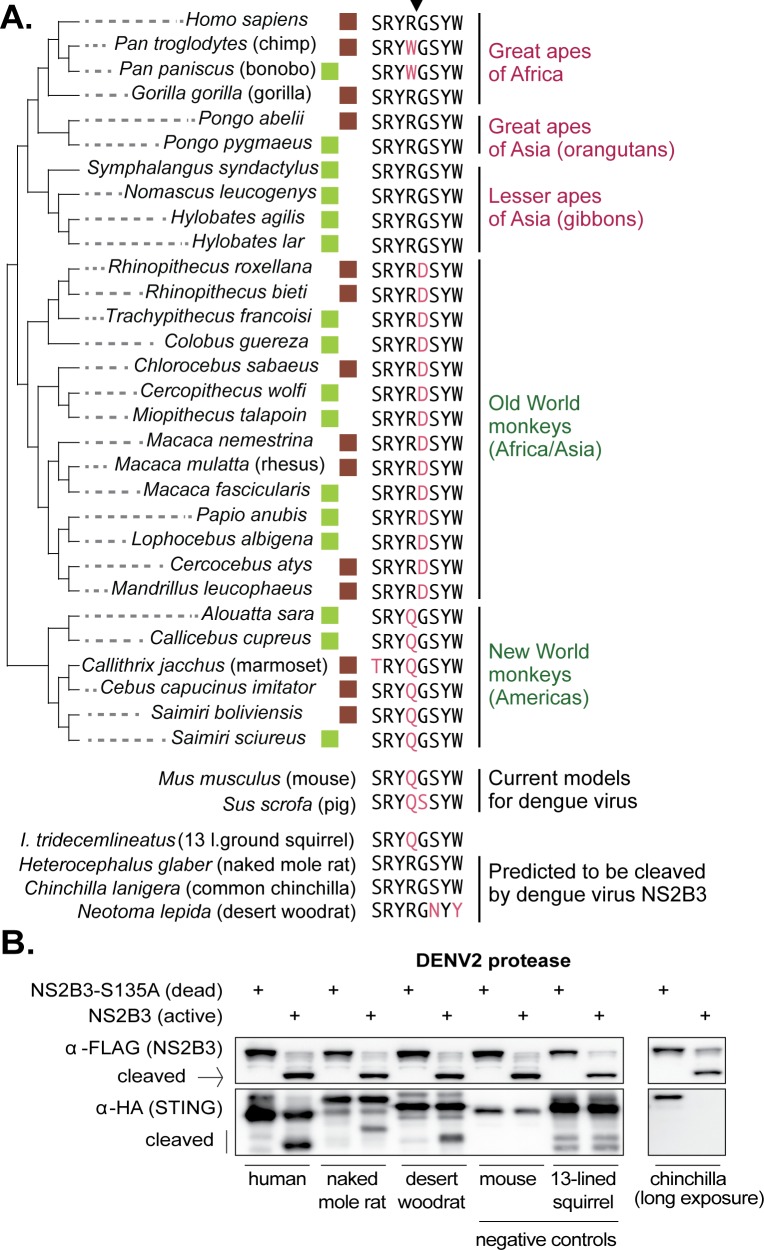
The dengue virus cleavage determinant in STING of various species. (**A**) An alignment of the eight amino acid region in STING surrounding residues 78R/79G, the newly identified dengue virus cleavage determinant (downward arrow at top). Deviations from the human motif are highlighted. The green boxes indicate STING orthologs sequenced as part of this study. The brown boxes indicate STING sequences obtained from Genbank. Apes are shown at the top of the tree (pink type), monkeys at the bottom (green type). Depicted below are sequences from the same region of STING from two animal models for dengue virus (mouse, pig [Cassetti et al., 2010]), several small rodent species which encode the correct cleavage motif at 78/79 (naked mole rat, common chinchilla, desert woodrat), and one that does not (13 lined ground squirrel). Genbank accession numbers of sequences shown: mouse (XP_017173483), pig (XP_005661761), 13-lined ground squirrel (XM_005327275), naked mole rat (JAO02071), chinchilla (XP_005382124), and desert woodrat (OBS58238). (**B**) STING-HA genes were synthesized for the rodent species discussed in panel A. Cleavage assays were performed by co-transfecting plasmids encoding the dengue protease (dead or active) as well as each STING, and then performing immunoblotting as described in the methods. The data presented are representative of at least two independent experiments.

It is curious to find that a sylvatic dengue virus does not cleave nonhuman primate STING. Since we don’t know the exact species that constitute the viral reservoir, we next considered the question of whether any nonhuman primates encode the correct cleavage determinant at positions 78/79 in STING. To address this, we harvested mRNA from cell lines derived from 16 different nonhuman primate species (see Materials and methods). From these mRNA pools, we made cDNA libraries and sequenced the STING cDNA using Sanger sequencing. We also gathered STING sequence for 14 additional primate species from Genbank. An alignment of the eight amino acid region in STING surrounding the 78/79 cleavage determinant (downward arrow in Figure 6A) is shown for all 30 of these primate species (a full alignment of primate STING sequences is provided in Supplementary file 1). With the exception of chimpanzees and bonobos, all apes encode the same amino acids as human in this motif, constituting the correct cleavage determinant for dengue virus. In contrast, no monkey species encodes an ‘RG’ at positions 78/79. Instead, Old World monkeys all encode ‘RD,’ which is the same motif found in the macaque clone that we have tested. Also, no monkeys from the Americas encode an ‘RG’ at these residues, and instead these species encode a ‘QG’ at positions 78/79, just like the marmoset clone tested herein.

Finally, we queried the entire Genbank database for STING sequences available from placental mammals. Mice and pigs, two current models for dengue virus infection (Cassetti et al., 2010), also do not have the correct RG residues at STING 78/79 (Figure 6A). Out of the entire database, only two other mammals were identified that share the exact same sequence as humans in the eight amino acid region surrounding the newly identified dengue virus cleavage determinant in STING: chinchilla and naked mole rat, both of which are rodents (Figure 6A). A third rodent species, the desert woodrat, has the RG at positions 78/79, but encodes two amino acid substitutions just downstream of these residues, compared to human STING (Figure 6A). The fact that only a small handful of mammals encode an RG at position 78/79, out of the entire database, may in part explain why modeling dengue viruses in animals has been so difficult. We next wished to determine if STING is in fact cleaved by dengue in these rodent species, since all three already serve as animal models for biological research (Keane et al., 2014; Nathaniel et al., 2013; Shimoyama et al., 2016; Campbell et al., 2016; Skopec et al., 2013). We synthesized HA-tagged STING genes for the rodent species discussed in Figure 6A, as well as an additional rodent (13-lined ground squirrel) which does not have the ‘RG’ motif at STING 78/79, as a negative control. Cleavage assays were performed using the co-transfection assay described previously. We see that the STING of naked mole rat and desert woodrat is clearly cleaved by the DENV2 protease, in that the cleavage product is evident (Figure 6B). We do not see a cleavage product for chinchilla STING, even under long exposure, but we do see the STING band disappear. It’s possible that, in this case, the cleavage product is too unstable to be detected. The identification of animal models encoding STING proteins that can be cleaved by dengue might be important; the advantage to using such species as models is that, unlike in STING knockout mice, the STING pathway would be intact in these animals.

## Discussion

In humans cells, sylvatic and human dengue viruses replicate similarly (Vasilakis et al., 2007, 2008). These results have been interpreted to mean that there is little or no adaptive barrier for the emergence of sylvatic dengue viruses into human populations. Our data agree with, but add a new element to, this model. Rather than there being critical differences between human and sylvatic viruses, our data suggest that there are critical differences between human and monkey hosts. This difference tracks, at least in part, to STING, revealing one way in which dengue viruses are reaching higher titers in humans than in monkey models. Collectively our data suggest that all dengue viruses cleave human STING, but not the versions of STING found in most other mammals. We have used the STING proteins of closely related primate species to map the determinant of cleavage in STING. We find that the viral protease is recognizing (i.e. cutting or binding) residues around positions 78/79 of STING. We show that an ‘RG’ motif at these two residues is necessary and sufficient for cleavage by the proteases of all four human epidemic dengue viruses, and one sylvatic dengue virus. Yet, only some apes and three rodent species, out of all of the mammalian STING sequences in Genbank, encode an RG at positions 78/79.

Why do dengue viruses universally cleave human but not monkey STING? It’s possible that what we have uncovered is a brilliant method for balancing alternate host species, one of which is dense and abundant (humans), versus others that are spare and exist in smaller populations (primates in nature). In this scenario, dengue viruses have evolved to suppress innate immunity in humans in order to increase viral titers and spread, even though this trait comes at the cost of increased pathogenicity in some individuals. This might be a good strategy in our abundant and dense host population, where the fitness cost of severe disease in a fraction of individuals would be outweighed by excellent spread. Remarkably, though, dengue viruses have achieved this by evolving to recognize a portion of human STING that is not conserved in the STING of the wild and more rare animals that serve as their sustaining reservoir in nature, allowing the viruses to maintain decreased pathogenicity in these species. The evolution of the viral proteases to cleave human STING and simultaneously to avoid cleavage of monkey STING would be expected to reduce virus titers in monkeys, as the interferon pathway would be at least partially enabled. This may be beneficial for many reasons, one of which is that the production of a low-level innate immune response may allow the virus to replicate in reservoir host species without inducing high titers and strong adaptive immune responses. Alternately, a second possibility is that sylvatic dengue viruses do cleave the STING of monkeys, but that the sylvatic virus (DakAr-141069) that we tested is not representative. However, we find this unlikely. Because DENV1-4 also cannot cleave monkey STING, and all derive from the sylvatic reservoir, this supports the finding that sylvatic viruses do not cleave monkey STING. Third, a final possibility is that apes are critical reservoirs for dengue viruses in nature. Gorillas encode ‘RG’ at 78/79 and are found in Africa, while orangutans and gibbons are found in Asia and also encode the correct cleavage motif for the dengue virus protease. In fact, wild orangutans have previously been found to have neutralizing antibodies against dengue virus (Wolfe et al., 2001). While apes could be playing a role as sylvatic hosts, the highly endangered and rare status of most apes makes it hard to believe that they are playing a major role in sustaining sylvatic dengue virus currently (Geissmann, 2007; Walsh et al., 2003).

The specific primate species that serve as the sustaining reservoir for sylvatic dengue viruses in nature are unknown (Vasilakis et al., 2011). Various Old World monkey species in both Asia and Africa are suspected hosts (Vasilakis et al., 2011; Rodhain, 1991; Diallo et al., 2003). For instance, sylvatic dengue viruses have been isolated directly from macaques (*Macaca fascicularis)* and leaf monkeys (*Presbytis obscura*) that were placed as sentinels in forest canopies (Rudnick, 1986). Other primates have been shown to have antibodies to dengue virus, including macaques (*Macaca fascicularis* and *Macaca nemestrina*), leaf monkeys (*Presbytis cristata*) (Rudnick, 1965), African green monkeys (*Chlorocebus sabaeus*) (Diallo et al., 2003), and one ape species, the Bornean orangutan (*Pongo pygmaeus*) (Wolfe et al., 2001). But these results are not definitive due to the cross-reactivity of antibodies directed against various flaviviruses (Calisher et al., 1989; Tesh et al., 2002; Mansfield et al., 2011), and the possibility that some primates might be accidental, rather than sustaining reservoir hosts (Vasilakis et al., 2011). Instead of being directly isolated from primates, most sylvatic dengue viruses have been obtained from forest mosquitoes (Diallo et al., 2003; Rudnick, 1986), or from humans that contracted the virus in the forest (Pyke et al., 2016; Cardosa et al., 2009; Franco et al., 2011). Therefore, there are many deficiencies in our understanding of the natural reservoir for dengue viruses. Interestingly, though, we have not identified any monkey species with an ‘RG’ at positions 78/79 in STING. Our results would indicate that dengue viruses, in general, cannot cleave the STING of monkey hosts. Our data suggests that even a sylvatic dengue virus, which we find targets the same residues in STING, would not be able to cleave STING of monkeys.

There are also implications of these findings to our understanding of dengue virus model organisms. If dengue proteases do not cleave most nonhuman forms of STING, this may at least partially explain why it has been so difficult to model dengue infection in immune-competent animals. Nonhuman primates infected with dengue virus generally don’t develop clinical signs of disease, consistent with enhanced control of the virus compared to humans (Cassetti et al., 2010). In fact, when human dengue viruses have been observed to replicate robustly in primate cell lines, these experiments have typically been done in cells such as Vero (Vasilakis et al., 2008; Rossi et al., 2012; Vasilakis et al., 2009) which are deficient in the type I interferon response (Osada et al., 2014; Desmyter et al., 1968). Human dengue viruses also cannot cleave mouse STING ((Aguirre et al., 2012; Yu et al., 2012) and herein), consistent with the heightened control of this virus in mice as well (Cassetti et al., 2010). Dengue virus will replicate to high titers in mice lacking key genes important for the interferon response, but for many reasons it is desirable to develop animal models in immune competent hosts (Cassetti et al., 2010). STING now adds to a growing list of host proteins that regulate viral infection differently even in closely related host species (for example, [Stabell et al., 2016; Lou et al., 2016; Rowley et al., 2016; Kerr et al., 2015; Meyerson et al., 2015; Demogines et al., 2012; Stremlau et al., 2004; Demogines et al., 2013; Ng et al., 2015; Hueffer et al., 2003; Radoshitzky et al., 2008; Patel et al., 2012; Elde et al., 2009; Martin et al., 2013; Miller et al., 2012; Sawyer and Elde, 2012; Meyerson et al., 2017]). The identification of such genes is critical to our understanding of viral adaptation during host switching, and to the development of animal models in which to study human viruses.

It is possible that the identification of small mammals that have a cleavage-susceptible STING would facilitate the development of better animal models for studying dengue virus. Our work suggests the identity of three such species: the naked mole rat, the common chinchilla, and the desert woodrat. All three of these small rodents are already used as animal models in biomedical research, and the genomes of all three have been sequenced (Keane et al., 2014; Nathaniel et al., 2013; Shimoyama et al., 2016; Campbell et al., 2016; Skopec et al., 2013). These species could be superior to STING knockout mice, in that the STING pathway would be intact and the cleavage of STING by the virus would be naturally modeled rather than just bypassed. These species may also be superior to future models where mouse (*Mus musculus*) STING would be replaced with human STING in transgenic animals. In this case, it is unknown if human STING would perform all of its functions the same in mouse as it does in humans. The advantage of using a rodent model with a STING that is naturally susceptible to dengue virus cleavage would be that the STING pathways would all be fully functional and intact. It is important to point out that, in addition to cleaving STING, dengue viruses modulate the interferon response in other ways as well. For instance, dengue viruses also bypass the type I interferon response by binding and degrading host STAT2 via the viral NS5 protein (Ashour et al., 2009; Jones et al., 2005; Mazzon et al., 2009; Best, 2017). Ideally, human dengue viruses would also be able to bind and degrade STAT2 in newly developed models, as they do in humans. Further, dengue viruses neutralize both the type I and type II interferon responses in other ways as well (Perry et al., 2011; Shresta et al., 2004; Aguirre et al., 2017; Aguirre and Fernandez-Sesma, 2017). Other known host-virus interactions would also need to be characterized in any potential new model organism.

It is notable that chimpanzees and bonobos encode STINGs that are resistant to cleavage, while STINGs of all other apes are susceptible. These two species differ from other apes in encoding a ‘WG’ at 78/79 of STING rather than the ‘RG’ encoded by all other apes (Figure 6). Remarkably, it was previously found that the ‘W’ at position 78, destroying the dengue cleavage determinant, was fixed by positive natural selection in wild chimpanzee populations (Mozzi et al., 2015). Chimpanzees are not one of the suspected natural reservoirs of dengue virus, but chimpanzee ranges do co-occur with known human outbreaks and with sylvatic cycles (Figure 1—figure supplement 1). One model is that, as dengue virus spread through Africa, a SNP in chimpanzee STING (or the STING of the chimpanzee/bonobo ancestor) at position 78 started to experience strong selection because it provided protection against cleavage by dengue viruses. This would have driven a selective sweep in chimpanzee populations, causing this species to become less susceptible to viral infection. It has previously been proposed that evolutionary pressure imposed by flavivirus proteases can drive selection at cleavage sites. For examples, the hepatitis C protease cleaves MAVS, another host signaling protein in the interferon induction cascade (Li et al., 2005b; Meylan et al., 2005). MAVS has experienced positive selection at a residue in the cleavage site for the hepatitis C virus protease (Patel et al., 2012). The authors of this study speculated that ancient viruses may have exerted selective pressure on primate genomes to acquire mutations in the cleavage site.

Like other viruses, dengue viruses remodel their host cellular environment in numerous ways, including the cleavage of STING and degradation of STAT2. Using the rich information that exists on how dengue viruses accomplish this, the genetic susceptibility of both suspected reservoir hosts, and potential new animal models, can be systematically assessed. Characterizing how host-virus interactions play out uniquely in different host species will help us to understand dengue virus in critical ways. For instance, it will reveal how dengue viruses do (or do not) need to evolve their genomes as they transmit to humans from nonhuman primates in nature. Also, understanding the genetics of host tropism will help identify better laboratory animals that can be used to study dengue virus pathogenesis and to develop drugs and vaccines.

## Materials and methods

**Key resources table:** 

Reagent type (species) or resource	Designation	Source or reference	Identifiers	Additional information
gene (Homo sapiens)	STING; TMEM173	NA	GENBANK:NM_198282	GENBANK:MF622062
gene (Homo sapiens)	STING; TMEM173	this study		GENBANK:MF622062
gene (Pan troglodytes)	STING; TMEM173	NA	GENBANK:XM_016953921	
gene (Pan paniscus)	STING; TMEM173	this study		GENBANK:MF616339
gene (Gorilla gorilla)	STING; TMEM173	NA	GENBANK:XM_0040426	
gene (Pongo abelii)	STING; TMEM173	NA	GENBANK:XM_002815952	
gene (Hylobates agilis)	STING; TMEM173	this study		GENBANK:MF616342
gene (Symphalangus syndactylus)	STING; TMEM173	this study		GENBANK:MF616343
gene (Nomascus leucogenys)	STING; TMEM173	this study		GENBANK:MF616344
gene (Hylobates lar)	STING; TMEM173	this study		GENBANK:MF616341
gene (Rhinopithecus roxellana)	STING; TMEM173	NA	GENBANK:XM_010388119	
gene (Rhinopithecus bieti)	STING; TMEM173	NA	GENBANK:XM_017895026	
gene (Trachypithecus francoisi)	STING; TMEM173	this study		GENBANK:MF616352
gene (Colobus guereza)	STING; TMEM173	this study		GENBANK:MF616351
gene (Chlorocebus sabaeus)	STING; TMEM173	NA	GENBANK:XM_008014636	
gene (Cercopithecus wolfi)	STING; TMEM173	this study		GENBANK:MF616350
gene (Miopithecus talapoin)	STING; TMEM173	this study		GENBANK:MF616349
gene (Macaca nemestrina)	STING; TMEM173	NA	GENBANK:XM_011716377	
gene (Macaca mulatta)	STING; TMEM173	NA	GENBANK:XM_015141010	
gene (Macaca mulatta)	STING; TMEM173	this study		GENBANK:MF622060
gene (Macaca fascicularis)	STING; TMEM173	this study		GENBANK:MF616346
gene (Papio papio)	STING; TMEM173	this study		GENBANK:MF616348
gene (Lophocebus albigena)	STING; TMEM173	this study		GENBANK:MF616347
gene (Cercocebus atys)	STING; TMEM173	NA	GENBANK:XM_012090448	
gene (Mandrillus leucophaeus)	STING; TMEM173	NA	GENBANK:XM_011997224	
gene (Aloutta sara)	STING; TMEM173	this study		GENBANK:MF616355
gene (Callicebus cupreus)	STING; TMEM173	this study		GENBANK:MF616354
gene (Callithrix jacchus)	STING; TMEM173	NA	GENBANK:XM_00898588	
gene (Callithrix jacchus)	STING; TMEM173	this study		GENBANK:MF622061
gene (Cebus capucinus imitator)	STING; TMEM173	NA	GENBANK:XM_017536735	
gene (Samiri boliviensis)	STING; TMEM173	NA	GENBANK:XM_003933913	
gene (Saimiri sciureus)	STING; TMEM173	this study		GENBANK:MF616353
gene (Mus musculus)	STING; TMEM173	NA	GENBANK:NM_001289591	
gene (Sus scrofa)	STING; TMEM173	NA	GENBANK:XP_005661761	
gene (Heterocephalus glaber)		NA	GENBANK:JAO02071	
gene (Chinchilla lanigera)		NA	GENBANK:XP_005382124	
gene (Neotoma lepida)		NA	GENBANK:OBS58238	
gene (Dengue viurs 2)	NS2B3	NA	GENBANK:M29095	
cell line (Homo sapiens)	293T cells	ATCC	CRL-3216	
cell line (Homo sapiens)	A549 cells	ATCC	CCL-185	
antibody	Rat anti-HA-HRP (3F10)	Sigma	11867423001	
antibody	Mouse anti-Flag (M2)	Sigma	F3165	
antibody	Rabbit anti-pIRF3	abcam	ab76493	
antibody	Rabbit anti-IRF3	Santa Cruz Biotech	sc-9082	
antibody	Rabbit anti-GAPDH	Cell Signaling	14C10	
antibody	Rabbit anti-STING	abcam	ab92605	
antibody	Mouse anti-Actin (C4)	Santa Cruz Biotech	Sc47778	
recombinant DNA reagent	DENV2 NS2B3 WT (plasmid)	PMID: 1642612		Progenitors: DENV2 NGC (GENBANK:M29095), pCR3.1
recombinant DNA reagent	DENV2 NS2B3 S135A (plasmid)	PMID: 1642612		Progenitors: DENV2 NS2B3 WT pCR3.1 plasmid, SDM
recombinant DNA reagent	DENV1 (Hawaii) cDNA	this paper		Progenitors: World Reference Center for Emerging Viruses and Arboviruses (WRCEVA) Catalog number NR-4287
recombinant DNA reagent	DENV2 (New Guinea C) cDNA	this paper		Progenitors: World Reference Center for Emerging Viruses and Arboviruses (WRCEVA) Catalog number NR-4288
recombinant DNA reagent	DENV3 (Philippines/ H87/1956) cDNA	this paper		Progenitors: World Reference Center for Emerging Viruses and Arboviruses (WRCEVA) Catalog number NR-2771
recombinant DNA reagent	DENV4 (H241) cDNA	this paper		Progenitors: World Reference Center for Emerging Viruses and Arboviruses (WRCEVA) Catalog number NR-4289
recombinant DNA reagent	DENV1 (Hawaii) NS2B3 WT (plasmid)	this paper		Progenitors: DENV1 (Hawaii) cDNA, pCR3.1
recombinant DNA reagent	DENV2 (New Guinea C) NS2B3 WT (plasmid)	this paper		Progenitors: DENV2 (New Guinea C) cDNA, pCR3.1
recombinant DNA reagent	DENV3 (Philippines/ H87/1956) NS2B3 WT (plasmid)	this paper		Progenitors: DENV3 (Philippines/H87/1956) cDNA, pCR3.1
recombinant DNA reagent	DENV4 (H241) NS2B3 WT (plasmid)	this paper		Progenitors: DENV4 (H241) cDNA, pCR3.1
recombinant DNA reagent	Sylvatic (DakAr-141069) Dengue NS2B3 Protease (WT)	this paper		Progenitors: DakAr-141069 NS2B3 sequence (GenBank EF105389)
recombinant DNA reagent	Sylvatic (DakAr-141069) Dengue NS2B3 Protease (S135A)	this paper		Progenitors: Sylvatic (DakAr-141069) Dengue NS2B3 Protease (WT) SDM product
recombinant DNA reagent	human cDNA	this paper		Progenitors: A549 cell line (ATCC CCL-185)
recombinant DNA reagent	chimpanzee cDNA	this paper		Progenitors: Coriell PR00748
recombinant DNA reagent	rhesus macaque cDNA	this paper		Progenitors: Mm265-95
recombinant DNA reagent	marmoset cDNA	this paper		Progenitors: Coriell PR07404
recombinant DNA reagent	mouse cDNA	this paper		Progenitors: RNA extracted from mouse liver
recombinant DNA reagent	human STING-HA (plasmid)	this paper		Progenitors: human cDNA, pcDNA3.1 plasmid
recombinant DNA reagent	human STING-HA (plasmid)	this paper		Progenitors: human cDNA, pLPCX plasmid
recombinant DNA reagent	human STING(R78W)-HA (plasmid)	this paper		Progenitors: human STING-HA pcDNA3.1 SDM product
recombinant DNA reagent	human STING(R78W)-HA (plasmid)	this paper		Progenitors: human STING-HA pLPCX SDM product
recombinant DNA reagent	human STING(R79D)-HA (plasmid)	this paper		Progenitors: human STING-HA pcDNA3.1 SDM product
recombinant DNA reagent	human STING(R78Q)-HA (plasmid)	this paper		Progenitors: human STING-HA pcDNA3.1 SDM product
recombinant DNA reagent	chimpanzee STING-HA (plasmid)	this paper		Progenitors: chimpanzee cDNA, pcDNA3.1 plasmid
recombinant DNA reagent	chimpanzee STING-HA (plasmid)	this paper		Progenitors: chimpanzee cDNA, pLPCX plasmid
recombinant DNA reagent	chimpanzee STING(W78R)-HA (plasmid)	this paper		Progenitors: chimpanzee STING-HA pcDNA3.1 SDM product
recombinant DNA reagent	chimpanzee STING(W78R)-HA (plasmid)	this paper		Progenitors: chimpanzee STING-HA pLPCX SDM product
recombinant DNA reagent	rhesus macaque STING-HA (plasmid)	this paper		Progenitors: rhesus macaque cDNA, pcDNA3.1 plasmid
recombinant DNA reagent	rhesus macaque STING(D79G)-HA (plasmid)	this paper		Progenitors: rhesus macaque STING-HA pcDNA3.1 SDM product
recombinant DNA reagent	marmoset STING-HA (plasmid)	this paper		Progenitors: marmoset cDNA, pcDNA3.1 plasmid
recombinant DNA reagent	marmoset STING(Q78R)-HA (plasmid)	this paper		Progenitors: marmoset STING-HA pcDNA3.1 SDM product
recombinant DNA reagent	mouse STING-HA (plasmid)	this paper		Progenitors: mouse cDNA, pcDNA3.1 plasmid
recombinant DNA reagent	mouse STING(Q78R)-HA (plasmid)	this paper		Progenitors: mouse STING-HA pcDNA3.1 SDM product
recombinant DNA reagent	mouse STING(93LRRG96)-HA (plasmid)	this paper		Progenitors: mouse STING-HA pcDNA3.1 SDM product
recombinant DNA reagent	human STING-3xFLAG (plasmid)	this paper		Progenitors: human cDNA, pLPCX plasmid
recombinant DNA reagent	human STING(R78W)-3xFLAG (plasmid)	this paper		Progenitors: human STING-3xFLAG pLPCX SDM product
recombinant DNA reagent	human STING(G230A)-3xFLAG (plasmid)	this paper		Progenitors: human STING-3xFLAG pLPCX SDM product
recombinant DNA reagent	human STING (R78W, G230A)-3xFLAG (plasmid)	this paper		Progenitors: human STING-3xFLAG pLPCX SDM product
recombinant DNA reagent	chimpanzee STING-3xFLAG (plasmid)	this paper		Progenitors: chimpanzee cDNA, pLPCX plasmid
recombinant DNA reagent	chimpanzee STING (W78R)-3xFLAG (plasmid)	this paper		Progenitors: chimpanzee STING-3xFLAG pLPCX SDM product
recombinant DNA reagent	chimpanzee STING(A230G)-3xFLAG (plasmid)	this paper		Progenitors: chimpanzee STING-3xFLAG pLPCX SDM product
recombinant DNA reagent	chimpanzee STING(W78R, A230G)-3xFLAG (plasmid)	this paper		Progenitors: chimpanzee STING-3xFLAG pLPCX SDM product
recombinant DNA reagent	IFN-ß1-luc (plasmid)	PMID: 21512573		
recombinant DNA reagent	pRL-CMV (plasmid)	Promega: AF025843		Progenitors: pRL-null
commercial assay or kit	Dual-Glo Luciferase Assay System	Promega	Cat#E2920	
commercial assay or kit	Superscript III First-Strand Synthesis System	Thermo Scientific	Cat#18080051	
software, algorithm	MEGA7			http://www.megasoftware.net/
software, algorithm	ImageJ version 1.43u			http://rsb.info.nih.gov/ij
software, algorithm	Python 2.7.11			https://www.python.org
software, algorithm	Sequencher			https://www.genecodes.com

### Plasmids

DENV2 NS2B3, expressed from the pCR3.1 plasmid, was a gift from Yi-Ling Lin. This plasmid, and all DENV1-4 protease-expressing plasmids described below, include a 3x FLAG tag at the C-terminus of NS3. For the experiment where proteases from human dengue viruses DENV1-4 are compared, primers were designed at the 5’ end of NS2B

(DENV1: taagcaAAGCTTcaccATGAGTTGGCCCCTC,

DENV2: taagcaAAGCTTcaccATGAGCTGGCCACTAAATGA,

DENV3: taagcaAAGCTTcaccATGAGCTGGCCACTG,

DENV4: taagcaAAGCTTcaccATGTCTTGGCCCCTTAAC) and the 3’ end of NS3

(DENV1: TGCTTAgtcgacaTCTTCTTCCTGCTGCAAACTCTTTAAACTC,

DENV2: TGCTTAgtcgacaCTTTCTTCCAGCTGCAAACTCCTTG,

DENV3: TGCTTAgtcgacaCTTTCTGCCAGCTGCAAAATCCTTG,

DENV4: TGCTTAgtcgacaCTTTCTTCCACTGGCAAACTCCTTG) to amplify the NS2B + NS3 genomic region in one fragment. In this experiment, the protease from DENV2 was re-cloned so that the structure of the four protease clones was identical in all four cases. The PCR templates were cDNAs created from RNA obtained through the World Reference Center for Emerging Viruses and Arboviruses (WRCEVA) (Cat# NR-32847). DENV1 (Hawaii, NR-4287), DENV2 (New Guinea C, NR-4288), DENV3 (Philippines/H87/1956, NR-2771), and DENV4 (H241, NR-4289). The PCR products, and the plasmid containing the DENV2 protease mentioned above (gift from Yi-Ling Lin), were both digested with HindIII and Sal1. The PCR products were ligated into this plasmid and transformed into DH5α chemically competent *E.coli*. The sylvatic NS2B3 (DakAr141069) was synthesized (without an epitope tag) using the sequence information deposited on NCBI (Genbank accession EF105389). STING genes used for functional analysis were amplified from cDNA libraries constructed from the following cell lines: human (A549), chimpanzee/bonobo (STING sequence identical for these two species, clone amplified from Coriell, PR00748), rhesus macaque (Mm265-95, a gift from Welkin Johnson), marmoset (Coriell, PR07404), and mouse (generated from RNA extracted from whole liver). Either an HA or 3xFlag tag were engineered onto the 3' end of the gene sequences, separated from the coding sequence by a 3xGlycine-Alanine (GAGAGA) linker region (nucleotide sequence = GGTGCTGGTGCTGGTGCT). These sequences were cloned into the pcDNA3.1 expression vector with a 5' Kozak sequence (GCCACC). Rodent STING constructs were synthesized (Quintarabio) to include a Kozak sequence, C-terminal HA-tag, and flanking linkers that were used for Gibson cloning into the pLPCX mammalian expression plasmid.

### STING cleavage assays

293 T cells (mycoplasma negative) were grown at 37°C in DMEM supplemented with 10% FBS, Pen/Strep, and L-glutamine. 24 hr prior to transfection, cells were plated at a density of 4.5 × 10^5^ cells per well in a 12-well dish in antibiotic free media. Wells were transfected with 800 ng plasmid encoding STING and 800 ng plasmid encoding NS2B3 using TransIT 293 reagent (Mirus MIR 2704). For most experiments (Figures 1B, 3B, 5A and B), cells were treated with 10uM MG132 for 8 hr prior to harvesting for western blot.

### Western blotting

Cells were lysed in RIPA buffer supplemented with protease inhibitor (Roche, 4693159001). Protein concentration was calculated using the Bradford method. 10% 37.5:1 Acrylamide/Bisacrylamide gels were used to run 30 ug of whole cell lysate for each sample. Protein was transferred overnight at 30 volts onto a polyvinyl membrane. Blocking was performed with a 10% milk solution in tris-buffered saline supplemented with 0.1% TWEEN20. Primary antibodies used were used against HA (3f10 clone Sigma 11867423001), Flag (M2 clone Sigma F3165), GAPDH (CellSignaling 14C10), STING (Abcam 92605), actin (Santa Cruz Biotech Sc47778), and dengue virus NS3 (mouse polyclonal antibody raised against purified full-length NS3 from dengue 2 strain 16681 [Heaton et al., 2010]). Secondary antibodies used were goat-anti-mouse-HRP (Thermo 62–6520) and goat-anti-rabbit (Thermo 65–6120). Blots were developed using ECL Prime (Amersham RPN2232) and imaged using ImagQuant LAS 4000 (Amersham 28-9558-10).

### CRISPR-Cas9 mediated disruption of STING, and stable re-complementation with primate orthologs

A549 cells (mycoplasma negative) were transfected with the pSPCAS9(BB)-P2A-eGFP (PX458) with the guide RNA sequence 5’ AGAGCACACTCTCCGGTACC 3’. GFP-positive cells were single-cell sorted into a 96-well dish and colonies were grown up. Cloned A549 cells were screened for homozygous mutations that disrupted the coding sequence of STING as follows. 10,000 cells were used to prep whole genomic DNA. The region surrounding the guide RNA was amplified using the following primers: 5’ GTCCCCAAGGGTTCTTGGTT 3’ and 5’ AACCAGTCCCACTCCCAGTA 3’. Amplified genomic DNA was Sanger sequenced to determine the nature of the CRISPR-CAS9-mediated genomic disruption. A cell line with confirmed homozygous disruption of STING (Figure 2—figure supplement 1) was then re-complemented with primate orthologs of STING. Four different C-terminally HA-tagged versions of STING were cloned into the pLPCX retroviral vector: wildtype human STING, R78W human STING, wildtype chimpanzee STING, and W78R chimpanzee STING. These were packaged into retroviral particles by cotransfecting into 293T cells (mycoplasma negative) each pLPCX-STING construct with plasmids expressing NB-tropic murine leukemia virus (MLV) Gag-Pol and VSV-G. As a control, we also made virus to complement with an empty pLPCX vector. Supernatants were collected and used to transduce 10^5 A549 cells in the presence of 10 ug/mL polybrene. 24 hr post transduction, cells were selected in 0.75 ug/mL puromycin.

### Immunofluorescence

24 hr after plating, cells were fixed with 4% paraformaldehyde and permeabilized with 1% TritonX100 in PBS. Blocking was performed with 3% BSA solution in PBS. Primary antibodies used were rabbit-anti-GRP78 (BiP) (Abcam ab21685) and mouse-anti-HA (clone 16B12 abcam ab130275). Secondary antibodies used were donkey-anti-rabbit conjugated to AlexaFluor594 (Invitrogen A21207) and donkey-anti-mouse conjugated to AlexaFluor488 (Invitrogen A21202). Cells were mounted using VECTASHIELD hardset mounting media (VectorLabs H-1400).

### Dengue infection assays

The indicated STING knockout and re-complemented cell lines were plated out in F-12K media with 10% FBS, after 24 hr the cells were counted. An MOI of 0.3 was calculated for each well and dengue virus 2 (16681) was allowed to attach to cells for 1 hr at room temperature. Unattached virus was then removed from cells, 2%serum in F-12K media was added to cells and they were maintained at 37°C with 5% CO_2_. After 24 and 48 hr the virus supernatant was removed for downstream titration on BHK21 cells. At the same time, cells were removed for downstream western blotting.

### Sequencing STING from other primate species

The following STING sequences were collected from GenBank: chimpanzee (*Pan troglodytes*, XM_016953921.1), gorilla (*Gorilla gorilla gorilla*, XM_004042612.1), Sumatran orangutan (*Pongo abelii*, XM_002815952.2), golden snub-nosed monkey (*Rhinopithecus roxellana*, XM_010388119.1), black snub-nosed monkey (*Rhinopithecus bieti*, XM_017895026.1), African green monkey (*Chlorocebus sabaeus*, XM_008014636.1), pigtail macaque (*Macaca nemestrina*, XM_011716377.1), rhesus macaque (*Macaca mulatta*, XM_015141010.1), sooty mangabey (*Cercocebus atys*, XM_012090448.1), drill (*Mandrillus leucophaeus*, XM_011997224.1), marmoset (*Callithrix jacchus*, XM_00898588.2), capuchin monkey (*Cebus capucinus imitator*, XM_017536735.1), black-capped squirrel monkey (*Saimiri boliviensis*, XP_003933962.1). The remaining STING gene sequences were obtained by direct sequencing of cDNA libraries produced from the following primary or immortalized primate fibroblast cell lines: Bonobo (*Pan* paniscus, Coriell PR00748), Bornean orangutan (*Pongo pygmaeus*, Coriell PR00650), white-handed gibbon (*Hylobates lar*, Coriell PR01131), agile gibbon (*Hylobates agilis*, Coriell PR00773), siamang (*Symphalagus syndactylus*, Coriell PR00722), white-cheeked gibbon (*Nomascus leucogenys*, Coriell PR01037), leaf monkey (*Trachypithecus francoisi*, Coriell PR01099), colobus monkey (*Colobus guereza*, Coriell PR00980), Wolf’s guenon (*Cercopithecus wolfi*, Coriell PR01241), talapoin (*Miopithecus talapoin*, Coriell PR00716), crab-eating macaque (*Macaca fasicularis*, 103–06, gift from Welkin Johnson), olive baboon (*Papio anubis*, Coriell PR00978), grey-cheeked mangabey (*Lophocebus albigena*, Coriell PR01215), Bolivian red howler monkey (*Alouatta sara*, Coriell PR00708), red titi monkey (*Callicebus* (or *Plecturocebus*) *cupreus*, Coriell PR00793), common squirrel monkey (*Saimiri sciureus*, Coriell PR00603). Briefly, cells were grown in DMEM (Cellgro) supplemented with 15% FBS (Gibco) at 37°C and 5% CO2. RNA was extracted using the AllPrep DNA/RNA extraction kit (QIAGEN). cDNA libraries were generated using SuperScript III first strand synthesis kit (Invitrogen). PCR was performed using PCR SuperMix High Fidelity (Invitrogen). PCR products were directly sequenced. Each primate sequence was used as a query to search the human genome, and human STING gene was returned as the top hit. STING gene sequences generated in this study have been deposited in GenBank (accession numbers MF616339-MF616355).

## Additional files

Supplementary file 1. Alignment of primate STING proteins.

Transparent reporting form

### Major datasets

The following dataset was generated:

Stabell A2018outbreak_mappinghttp://dx.doi.org/10.5061/dryad.pm7chAvailable at Dryad Digital Repository under a CC0 Public Domain Dedication

The following previously published dataset was used:

Messina JPBrady OJPigott DMBrownstein JSHoen AGHay SI2014Dengue_Occurrence_12122013.xlsxhttps://figshare.com/articles/Dengue_Occurrence_12122013.xlsx/1035043Available at figshare under a CC0 Public Domain licence

## References

[bib1] Aguirre S, Fernandez-Sesma A (2017). Collateral Damage during Dengue Virus Infection: Making Sense of DNA by cGAS. Journal of Virology.

[bib2] Aguirre S, Luthra P, Sanchez-Aparicio MT, Maestre AM, Patel J, Lamothe F, Fredericks AC, Tripathi S, Zhu T, Pintado-Silva J, Webb LG, Bernal-Rubio D, Solovyov A, Greenbaum B, Simon V, Basler CF, Mulder LC, García-Sastre A, Fernandez-Sesma A (2017). Dengue virus NS2B protein targets cGAS for degradation and prevents mitochondrial DNA sensing during infection. Nature Microbiology.

[bib3] Aguirre S, Maestre AM, Pagni S, Patel JR, Savage T, Gutman D, Maringer K, Bernal-Rubio D, Shabman RS, Simon V, Rodriguez-Madoz JR, Mulder LC, Barber GN, Fernandez-Sesma A (2012). DENV inhibits type I IFN production in infected cells by cleaving human STING. PLoS Pathogens.

[bib4] Althouse BM, Durbin AP, Hanley KA, Halstead SB, Weaver SC, Cummings DA (2014). Viral kinetics of primary dengue virus infection in non-human primates: a systematic review and individual pooled analysis. Virology.

[bib5] Ashour J, Laurent-Rolle M, Shi PY, García-Sastre A (2009). NS5 of dengue virus mediates STAT2 binding and degradation. Journal of Virology.

[bib6] Ashour J, Morrison J, Laurent-Rolle M, Belicha-Villanueva A, Plumlee CR, Bernal-Rubio D, Williams KL, Harris E, Fernandez-Sesma A, Schindler C, García-Sastre A (2010). Mouse STAT2 restricts early dengue virus replication. Cell Host & Microbe.

[bib7] Bera AK, Kuhn RJ, Smith JL (2007). Functional characterization of cis and trans activity of the Flavivirus NS2B-NS3 protease. Journal of Biological Chemistry.

[bib8] Best SM (2016). Flaviviruses. Current Biology.

[bib9] Best SM (2017). The Many Faces of the Flavivirus NS5 Protein in Antagonism of Type I Interferon Signaling. Journal of Virology.

[bib10] Bhatt S, Gething PW, Brady OJ, Messina JP, Farlow AW, Moyes CL, Drake JM, Brownstein JS, Hoen AG, Sankoh O, Myers MF, George DB, Jaenisch T, Wint GR, Simmons CP, Scott TW, Farrar JJ, Hay SI (2013). The global distribution and burden of dengue. Nature.

[bib11] Burdette DL, Vance RE (2013). STING and the innate immune response to nucleic acids in the cytosol. Nature Immunology.

[bib12] Calisher CH, Karabatsos N, Dalrymple JM, Shope RE, Porterfield JS, Westaway EG, Brandt WE (1989). Antigenic relationships between flaviviruses as determined by cross-neutralization tests with polyclonal antisera. Journal of General Virology.

[bib13] Campbell M, Oakeson KF, Yandell M, Halpert JR, Dearing D (2016). The draft genome sequence and annotation of the desert woodrat Neotoma lepida. Genomics Data.

[bib14] Cardosa J, Ooi MH, Tio PH, Perera D, Holmes EC, Bibi K, Abdul Manap Z (2009). Dengue virus serotype 2 from a sylvatic lineage isolated from a patient with dengue hemorrhagic fever. PLoS Neglected Tropical Diseases.

[bib15] Cassetti MC, Durbin A, Harris E, Rico-Hesse R, Roehrig J, Rothman A, Whitehead S, Natarajan R, Laughlin C (2010). Report of an NIAID workshop on dengue animal models. Vaccine.

[bib16] Chambers TJ, McCourt DW, Rice CM (1990). Production of yellow fever virus proteins in infected cells: identification of discrete polyprotein species and analysis of cleavage kinetics using region-specific polyclonal antisera. Virology.

[bib17] Demogines A, Abraham J, Choe H, Farzan M, Sawyer SL (2013). Dual host-virus arms races shape an essential housekeeping protein. PLoS Biology.

[bib18] Demogines A, Farzan M, Sawyer SL (2012). Evidence for ACE2-utilizing coronaviruses (CoVs) related to severe acute respiratory syndrome CoV in bats. Journal of Virology.

[bib19] Desmyter J, Melnick JL, Rawls WE (1968). Defectiveness of interferon production and of rubella virus interference in a line of African green monkey kidney cells (Vero). Journal of Virology.

[bib20] Diallo M, Ba Y, Sall AA, Diop OM, Ndione JA, Mondo M, Girault L, Mathiot C (2003). Amplification of the sylvatic cycle of dengue virus type 2, Senegal, 1999-2000: entomologic findings and epidemiologic considerations. Emerging Infectious Diseases.

[bib21] Diamond MS, Pierson TC (2015). Molecular Insight into Dengue Virus Pathogenesis and Its Implications for Disease Control. Cell.

[bib22] Ding Q, Cao X, Lu J, Huang B, Liu YJ, Kato N, Shu HB, Zhong J (2013). Hepatitis C virus NS4B blocks the interaction of STING and TBK1 to evade host innate immunity. Journal of Hepatology.

[bib23] Elde NC, Child SJ, Geballe AP, Malik HS (2009). Protein kinase R reveals an evolutionary model for defeating viral mimicry. Nature.

[bib24] Falgout B, Pethel M, Zhang YM, Lai CJ (1991). Both nonstructural proteins NS2B and NS3 are required for the proteolytic processing of dengue virus nonstructural proteins. Journal of Virology.

[bib25] Ferreira MS, de Castro PH, Silva GA, Casseb SM, Dias Júnior AG, Rodrigues SG, Azevedo RS, Costa e Silva MF, Zauli DA, Araújo MS, Béla SR, Teixeira-Carvalho A, Martins-Filho OA, Vasconcelos PF (2014). Callithrix penicillata: a feasible experimental model for dengue virus infection. Immunology Letters.

[bib26] Franco L, Palacios G, Martinez JA, Vázquez A, Savji N, De Ory F, Sanchez-Seco MP, Martín D, Lipkin WI, Tenorio A (2011). First report of sylvatic DENV-2-associated dengue hemorrhagic fever in West Africa. PLoS Neglected Tropical Diseases.

[bib27] Freifeld CC, Mandl KD, Reis BY, Brownstein JS (2008). HealthMap: global infectious disease monitoring through automated classification and visualization of Internet media reports. Journal of the American Medical Informatics Association.

[bib28] Geissmann T (2007). Status reassessment of the gibbons: Results of the Asian primate red list workshop 2006. Gibbon Journal.

[bib29] Green AM, Beatty PR, Hadjilaou A, Harris E (2014). Innate immunity to dengue virus infection and subversion of antiviral responses. Journal of Molecular Biology.

[bib30] Halstead SB, Shotwell H, Casals J (1973). Studies on the pathogenesis of dengue infection in monkeys. I. Clinical laboratory responses to primary infection. Journal of Infectious Diseases.

[bib31] Hanley KA, Monath TP, Weaver SC, Rossi SL, Richman RL, Vasilakis N (2013). Fever versus fever: the role of host and vector susceptibility and interspecific competition in shaping the current and future distributions of the sylvatic cycles of dengue virus and yellow fever virus. Infection, Genetics and Evolution.

[bib32] Heaton NS, Perera R, Berger KL, Khadka S, Lacount DJ, Kuhn RJ, Randall G (2010). Dengue virus nonstructural protein 3 redistributes fatty acid synthase to sites of viral replication and increases cellular fatty acid synthesis. PNAS.

[bib33] Hickey AC, Koster JA, Thalmann CM, Hardcastle K, Tio PH, Cardosa MJ, Bossart KN (2013). Serotype-specific host responses in rhesus macaques after primary dengue challenge. The American Journal of Tropical Medicine and Hygiene.

[bib34] Holm CK, Rahbek SH, Gad HH, Bak RO, Jakobsen MR, Jiang Z, Hansen AL, Jensen SK, Sun C, Thomsen MK, Laustsen A, Nielsen CG, Severinsen K, Xiong Y, Burdette DL, Hornung V, Lebbink RJ, Duch M, Fitzgerald KA, Bahrami S, Mikkelsen JG, Hartmann R, Paludan SR (2016). Influenza A virus targets a cGAS-independent STING pathway that controls enveloped RNA viruses. Nature Communications.

[bib35] Hueffer K, Parker JS, Weichert WS, Geisel RE, Sgro JY, Parrish CR (2003). The natural host range shift and subsequent evolution of canine parvovirus resulted from virus-specific binding to the canine transferrin receptor. Journal of Virology.

[bib36] Ishikawa H, Barber GN (2008). STING is an endoplasmic reticulum adaptor that facilitates innate immune signalling. Nature.

[bib37] Ishikawa H, Ma Z, Barber GN (2009). STING regulates intracellular DNA-mediated, type I interferon-dependent innate immunity. Nature.

[bib38] Jin L, Waterman PM, Jonscher KR, Short CM, Reisdorph NA, Cambier JC (2008). MPYS, a novel membrane tetraspanner, is associated with major histocompatibility complex class II and mediates transduction of apoptotic signals. Molecular and Cellular Biology.

[bib39] Jones M, Davidson A, Hibbert L, Gruenwald P, Schlaak J, Ball S, Foster GR, Jacobs M (2005). Dengue virus inhibits alpha interferon signaling by reducing STAT2 expression. Journal of Virology.

[bib40] Keane M, Craig T, Alföldi J, Berlin AM, Johnson J, Seluanov A, Gorbunova V, Di Palma F, Lindblad-Toh K, Church GM, de Magalhães JP (2014). The Naked Mole Rat Genome Resource: facilitating analyses of cancer and longevity-related adaptations. Bioinformatics.

[bib41] Kerr SA, Jackson EL, Lungu OI, Meyer AG, Demogines A, Ellington AD, Georgiou G, Wilke CO, Sawyer SL (2015). Computational and Functional Analysis of the Virus-Receptor Interface Reveals Host Range Trade-Offs in New World Arenaviruses. Journal of Virology.

[bib42] Li J, Lim SP, Beer D, Patel V, Wen D, Tumanut C, Tully DC, Williams JA, Jiricek J, Priestle JP, Harris JL, Vasudevan SG (2005a). Functional profiling of recombinant NS3 proteases from all four serotypes of dengue virus using tetrapeptide and octapeptide substrate libraries. Journal of Biological Chemistry.

[bib43] Li XD, Sun L, Seth RB, Pineda G, Chen ZJ (2005b). Hepatitis C virus protease NS3/4A cleaves mitochondrial antiviral signaling protein off the mitochondria to evade innate immunity. PNAS.

[bib44] Lou DI, Kim ET, Meyerson NR, Pancholi NJ, Mohni KN, Enard D, Petrov DA, Weller SK, Weitzman MD, Sawyer SL (2016). An Intrinsically Disordered Region of the DNA Repair Protein Nbs1 Is a Species-Specific Barrier to Herpes Simplex Virus 1 in Primates. Cell Host & Microbe.

[bib45] Mansfield KL, Horton DL, Johnson N, Li L, Barrett AD, Smith DJ, Galbraith SE, Solomon T, Fooks AR (2011). Flavivirus-induced antibody cross-reactivity. Journal of General Virology.

[bib46] Martin C, Buckler-White A, Wollenberg K, Kozak CA (2013). The avian XPR1 gammaretrovirus receptor is under positive selection and is disabled in bird species in contact with virus-infected wild mice. Journal of Virology.

[bib47] Mayer SV, Tesh RB, Vasilakis N (2017). The emergence of arthropod-borne viral diseases: A global prospective on dengue, chikungunya and zika fevers. Acta Tropica.

[bib48] Mazzon M, Jones M, Davidson A, Chain B, Jacobs M (2009). Dengue virus NS5 inhibits interferon-alpha signaling by blocking signal transducer and activator of transcription 2 phosphorylation. The Journal of Infectious Diseases.

[bib49] Messina JP, Brady OJ, Pigott DM, Brownstein JS, Hoen AG, Hay SI (2014). A global compendium of human dengue virus occurrence. Scientific Data.

[bib50] Meyerson NR, Sharma A, Wilkerson GK, Overbaugh J, Sawyer SL (2015). Identification of Owl Monkey CD4 Receptors Broadly Compatible with Early-Stage HIV-1 Isolates. Journal of Virology.

[bib51] Meyerson NR, Zhou L, Guo YR, Zhao C, Tao YJ, Krug RM, Sawyer SL (2017). Nuclear TRIM25 Specifically Targets Influenza Virus Ribonucleoproteins to Block the Onset of RNA Chain Elongation. Cell Host & Microbe.

[bib52] Meylan E, Curran J, Hofmann K, Moradpour D, Binder M, Bartenschlager R, Tschopp J (2005). Cardif is an adaptor protein in the RIG-I antiviral pathway and is targeted by hepatitis C virus. Nature.

[bib53] Miller EH, Obernosterer G, Raaben M, Herbert AS, Deffieu MS, Krishnan A, Ndungo E, Sandesara RG, Carette JE, Kuehne AI, Ruthel G, Pfeffer SR, Dye JM, Whelan SP, Brummelkamp TR, Chandran K (2012). Ebola virus entry requires the host-programmed recognition of an intracellular receptor. The EMBO Journal.

[bib54] Moi ML, Omatsu T, Hirayama T, Nakamura S, Katakai Y, Yoshida T, Saito A, Tajima S, Ito M, Takasaki T, Akari H, Kurane I (2013). Presence of Viral Genome in Urine and Development of Hematuria and Pathological Changes in Kidneys in Common Marmoset (Callithrix jacchus) after Inoculation with Dengue Virus. Pathogens.

[bib55] Morrison J, Aguirre S, Fernandez-Sesma A (2012). Innate immunity evasion by Dengue virus. Viruses.

[bib56] Mozzi A, Pontremoli C, Forni D, Clerici M, Pozzoli U, Bresolin N, Cagliani R, Sironi M (2015). OASes and STING: adaptive evolution in concert. Genome Biology and Evolution.

[bib57] Nathaniel TI, Otukonyong EE, Okon M, Chaves J, Cochran T, Nathaniel AI (2013). Metabolic regulatory clues from the naked mole rat: toward brain regulatory functions during stroke. Brain Research Bulletin.

[bib58] Ng M, Ndungo E, Kaczmarek ME, Herbert AS, Binger T, Kuehne AI, Jangra RK, Hawkins JA, Gifford RJ, Biswas R, Demogines A, James RM, Yu M, Brummelkamp TR, Drosten C, Wang LF, Kuhn JH, Müller MA, Dye JM, Sawyer SL, Chandran K (2015). Filovirus receptor NPC1 contributes to species-specific patterns of ebolavirus susceptibility in bats. eLife.

[bib59] Nitta S, Sakamoto N, Nakagawa M, Kakinuma S, Mishima K, Kusano-Kitazume A, Kiyohashi K, Murakawa M, Nishimura-Sakurai Y, Azuma S, Tasaka-Fujita M, Asahina Y, Yoneyama M, Fujita T, Watanabe M (2013). Hepatitis C virus NS4B protein targets STING and abrogates RIG-I-mediated type I interferon-dependent innate immunity. Hepatology.

[bib60] Osada N, Kohara A, Yamaji T, Hirayama N, Kasai F, Sekizuka T, Kuroda M, Hanada K (2014). The genome landscape of the african green monkey kidney-derived vero cell line. DNA Research.

[bib61] Patel MR, Loo YM, Horner SM, Gale M, Malik HS (2012). Convergent evolution of escape from hepaciviral antagonism in primates. PLoS Biology.

[bib62] Perelman P, Johnson WE, Roos C, Seuánez HN, Horvath JE, Moreira MA, Kessing B, Pontius J, Roelke M, Rumpler Y, Schneider MP, Silva A, O'Brien SJ, Pecon-Slattery J (2011). A molecular phylogeny of living primates. PLoS Genetics.

[bib63] Perera R, Kuhn RJ (2008). Structural proteomics of dengue virus. Current Opinion in Microbiology.

[bib64] Perry ST, Buck MD, Lada SM, Schindler C, Shresta S (2011). STAT2 mediates innate immunity to Dengue virus in the absence of STAT1 via the type I interferon receptor. PLoS Pathogens.

[bib65] Preugschat F, Yao CW, Strauss JH (1990). In vitro processing of dengue virus type 2 nonstructural proteins NS2A, NS2B, and NS3. Journal of Virology.

[bib66] Pyke AT, Moore PR, Taylor CT, Hall-Mendelin S, Cameron JN, Hewitson GR, Pukallus DS, Huang B, Warrilow D, van den Hurk AF (2016). Highly divergent dengue virus type 1 genotype sets a new distance record. Scientific Reports.

[bib67] Radoshitzky SR, Kuhn JH, Spiropoulou CF, Albariño CG, Nguyen DP, Salazar-Bravo J, Dorfman T, Lee AS, Wang E, Ross SR, Choe H, Farzan M (2008). Receptor determinants of zoonotic transmission of New World hemorrhagic fever arenaviruses. PNAS.

[bib68] Rico-Hesse R (1990). Molecular evolution and distribution of dengue viruses type 1 and 2 in nature. Virology.

[bib69] Rodhain F (1991). The role of monkeys in the biology of dengue and yellow fever. Comparative Immunology, Microbiology and Infectious Diseases.

[bib70] Rodriguez-Madoz JR, Belicha-Villanueva A, Bernal-Rubio D, Ashour J, Ayllon J, Fernandez-Sesma A (2010). Inhibition of the type I interferon response in human dendritic cells by dengue virus infection requires a catalytically active NS2B3 complex. Journal of Virology.

[bib71] Rossi SL, Nasar F, Cardosa J, Mayer SV, Tesh RB, Hanley KA, Weaver SC, Vasilakis N (2012). Genetic and phenotypic characterization of sylvatic dengue virus type 4 strains. Virology.

[bib72] Rowley PA, Ho B, Bushong S, Johnson A, Sawyer SL (2016). XRN1 Is a Species-Specific Virus Restriction Factor in Yeasts. PLOS Pathogens.

[bib73] Rudnick A (1965). Studies of the ecology of dengue in Malaysia: a preliminary report. Journal of Medical Entomology.

[bib74] Rudnick A (1986). Dengue VIrus Ecology in Malasia. Inst Med Res Malays Bull.

[bib75] Sawyer SL, Elde NC (2012). A cross-species view on viruses. Current Opinion in Virology.

[bib76] Scherer WF, Russell PK, Rosen L, Casals J, Dickerman RW (1978). Experimental infection of chimpanzees with dengue viruses. The American Journal of Tropical Medicine and Hygiene.

[bib77] Scherwitzl I, Mongkolsapaja J, Screaton G (2017). Recent advances in human flavivirus vaccines. Current Opinion in Virology.

[bib78] Shimoyama M, Smith JR, De Pons J, Tutaj M, Khampang P, Hong W, Erbe CB, Ehrlich GD, Bakaletz LO, Kerschner JE (2016). The Chinchilla Research Resource Database: resource for an otolaryngology disease model. Database.

[bib79] Shresta S, Kyle JL, Snider HM, Basavapatna M, Beatty PR, Harris E (2004). Interferon-dependent immunity is essential for resistance to primary dengue virus infection in mice, whereas T- and B-cell-dependent immunity are less critical. Journal of Virology.

[bib80] Skopec MM, Malenke JR, Halpert JR, Denise Dearing M (2013). An in vivo assay for elucidating the importance of cytochromes P450 for the ability of a wild mammalian herbivore (Neotoma lepida) to consume toxic plants. Physiological and Biochemical Zoology.

[bib81] Stabell AC, Hawkins J, Li M, Gao X, David M, Press WH, Sawyer SL (2016). Non-human Primate Schlafen11 Inhibits Production of Both Host and Viral Proteins. PLOS Pathogens.

[bib82] Stremlau M, Owens CM, Perron MJ, Kiessling M, Autissier P, Sodroski J (2004). The cytoplasmic body component TRIM5alpha restricts HIV-1 infection in Old World monkeys. Nature.

[bib83] Sun L, Xing Y, Chen X, Zheng Y, Yang Y, Nichols DB, Clementz MA, Banach BS, Li K, Baker SC, Chen Z (2012). Coronavirus papain-like proteases negatively regulate antiviral innate immune response through disruption of STING-mediated signaling. PLoS One.

[bib84] Sun W, Li Y, Chen L, Chen H, You F, Zhou X, Zhou Y, Zhai Z, Chen D, Jiang Z (2009). ERIS, an endoplasmic reticulum IFN stimulator, activates innate immune signaling through dimerization. PNAS.

[bib85] Tesh RB, Travassos da Rosa AP, Guzman H, Araujo TP, Xiao SY (2002). Immunization with heterologous flaviviruses protective against fatal West Nile encephalitis. Emerging Infectious Diseases.

[bib86] Vasilakis N, Cardosa J, Hanley KA, Holmes EC, Weaver SC (2011). Fever from the forest: prospects for the continued emergence of sylvatic dengue virus and its impact on public health. Nature Reviews Microbiology.

[bib87] Vasilakis N, Deardorff ER, Kenney JL, Rossi SL, Hanley KA, Weaver SC (2009). Mosquitoes put the brake on arbovirus evolution: experimental evolution reveals slower mutation accumulation in mosquito than vertebrate cells. PLoS Pathogens.

[bib88] Vasilakis N, Fokam EB, Hanson CT, Weinberg E, Sall AA, Whitehead SS, Hanley KA, Weaver SC (2008). Genetic and phenotypic characterization of sylvatic dengue virus type 2 strains. Virology.

[bib89] Vasilakis N, Shell EJ, Fokam EB, Mason PW, Hanley KA, Estes DM, Weaver SC (2007). Potential of ancestral sylvatic dengue-2 viruses to re-emerge. Virology.

[bib90] Vasilakis N, Weaver SC (2008). The history and evolution of human dengue emergence. Advances in Virus Research.

[bib91] Walsh PD, Abernethy KA, Bermejo M, Beyers R, De Wachter P, Akou ME, Huijbregts B, Mambounga DI, Toham AK, Kilbourn AM, Lahm SA, Latour S, Maisels F, Mbina C, Mihindou Y, Obiang SN, Effa EN, Starkey MP, Telfer P, Thibault M, Tutin CE, White LJ, Wilkie DS (2003). Catastrophic ape decline in western equatorial Africa. Nature.

[bib92] Wang E, Ni H, Xu R, Barrett AD, Watowich SJ, Gubler DJ, Weaver SC (2000). Evolutionary relationships of endemic/epidemic and sylvatic dengue viruses. Journal of Virology.

[bib93] Weaver SC, Vasilakis N (2009). Molecular evolution of dengue viruses: contributions of phylogenetics to understanding the history and epidemiology of the preeminent arboviral disease. Infection, Genetics and Evolution.

[bib94] Wolfe ND, Kilbourn AM, Karesh WB, Rahman HA, Bosi EJ, Cropp BC, Andau M, Spielman A, Gubler DJ (2001). Sylvatic transmission of arboviruses among Bornean orangutans. The American Journal of Tropical Medicine and Hygiene.

[bib95] Wu X, Wu FH, Wang X, Wang L, Siedow JN, Zhang W, Pei ZM (2014). Molecular evolutionary and structural analysis of the cytosolic DNA sensor cGAS and STING. Nucleic Acids Research.

[bib96] Yu CY, Chang TH, Liang JJ, Chiang RL, Lee YL, Liao CL, Lin YL (2012). Dengue virus targets the adaptor protein MITA to subvert host innate immunity. PLoS Pathogens.

[bib97] Yusof R, Clum S, Wetzel M, Murthy HM, Padmanabhan R (2000). Purified NS2B/NS3 serine protease of dengue virus type 2 exhibits cofactor NS2B dependence for cleavage of substrates with dibasic amino acids in vitro. Journal of Biological Chemistry.

[bib98] Zhang L, Mohan PM, Padmanabhan R (1992). Processing and localization of Dengue virus type 2 polyprotein precursor NS3-NS4A-NS4B-NS5. Journal of Virology.

[bib99] Zhang Z, Yuan B, Bao M, Lu N, Kim T, Liu YJ (2011). The helicase DDX41 senses intracellular DNA mediated by the adaptor STING in dendritic cells. Nature Immunology.

[bib100] Zhong B, Yang Y, Li S, Wang YY, Li Y, Diao F, Lei C, He X, Zhang L, Tien P, Shu HB (2008). The adaptor protein MITA links virus-sensing receptors to IRF3 transcription factor activation. Immunity.

[bib101] Zompi S, Harris E (2012). Animal models of dengue virus infection. Viruses.

