# Supplementary material for: Dengue viruses cleave STING in humans but not in nonhuman primates, their presumed natural reservoir
Source: eLife. 2018 Mar 20;7:e31919. doi: 10.7554/eLife.31919 (PMC5860865; doi:10.7554/eLife.31919)
Supplement: Supplementary file 1. [file elife-31919-supp1.docx]

**Supplemental Figure S6. Alignment of primate STING proteins**

Chimp MPHSSLHPSIPCPRGHGAQKAALVLLSACLVTLWGLGEPPEHTLRYLVLHLASLQLGLLL

Bonobo MPHSSLHPSIPCPRGHGAQKAALVLLSACLVTLWGLGEPPEHTLRYLVLHLASLQLGLLL

Human MPHSSLHPSIPCPRGHGAQKAALVLLSACLVTLWGLGEPPEHTLRYLVLHLASLQLGLLL

Gggorilla MPHSSLHPSIPCPRGHGAQKAALVLLSACLVTLWGLGEPPEHTLRCLVLHLASLQLGLLL

Borang MPHSSLHPSIPCPRGHGAQKAALVLLSACLVTLWGLGEPPEHTLQCLVLHLASLQLGLLL

P_abelii MPHSSLHPSIPCPRGHGAQKAALVLLSACLVTLWGLGEPPEHTLQCLVLHLASLQLGLLL

WHgib MPHSSLHPSIPCPRGHGAQKAALALLGACLVTLWGLGEPAEHTLRCLVLHLASLQLGLLL

AgileGib MPHSSLHPSIPCPRGHGAQKAALALLGACLVTLWGLGEPAEHTLRCLVLHLASLQLGLLL

Siamang MPHSSLHPSIPCPRGHGAQKAALVLLGACLVTLWGLGEPAEHTLRCLVLHLASLQLGLLL

WCgib MPHSSLHPSIPCLRGHGAQKAALVLLGACLVTLWGLGEPAEHTLRCLVLQLASLQLGLLL

Mleucoph MTRSSLHPSIPCPRGHGAQKAALVLLTACLGTLWGLGESPEHILRCLVFHLASLQLGQLL

C_atys MTRSSLHPSIPCPRGHGAQKAALVLLTACLGTLWGLGESPEHILRCLVFHLASLQLGQLL

P_anubis MTRSSLHPSIPCPRGHGAQKAALVLLTACLGTLWGLGESPEHILRCLVFHLASLQLGQLL

BlackMan MTRSSLHPSIPCPRGHGAQKAALVLLTACLGTLWGLGESPEHILRCLVFHLASLQLGQLL

Mnem MTRSSLHPSIPCPRGHGAQKAALVLLTACLGTLWGLGESPEHILRCLVLHLASLQLGQLL

Mfascic MTRSSLHPSIPCPRGHGAQKAALVLLTACLGTLWGLGESPEHILRCLVLHLASLQLGQLL

Mmull MTRSSLHPSIPCPRGHGAQKAALVLLTACLGTLWGLGESPEHILRCLVLHLASLQLGQLL

Talapoin MTRSSLHPSIPCPRGHGAQKAALVLLTACLVTLWGLGESPEHILRCLVFHLASLQLGQLL

WolfG MTRSSLHPSIPCPRGHGAQKAALVLLTACLVTLWGLGESPEHILRCLVLHLASLQLGQLL

Chlor_sab MTRSSLHPSIPCPRGHGAQKAALVLLTACLVTLWGLGESPEHILRCLVLHLASLQLGQLL

Rbieti MPRSSLHPSIPCPRGHGAQKAALVLLTACLGTLWGLGESPEHILRCLVLHLASLQLGQLL

Rroxell MPRSSLHPSIPCPRGHGAQKAALVLLTACLGTLWGLGESPEHILRCLVLHLASLQLGQLL

LeafM MPRSSLHPSIPCPRGHGAQKAALVLLTACLGTLWGLGESPEHILRCLVLHLASLQLGQLL

Colobus MPRSSLHPSIPCPRGHGAQKAALVLLTACLGTLWGLGESPEHTLRCLVLHLASLQLGQLL

SquirrelM MPHSSLHPSIPHLRGHGTQKAALVLLNVCLVTLWWLGEPAEDILQFLVLHLASLQLGLLL

Sbboliv MPHSSLHPSIPHPRGHGTQKAALVLLNVCLVTLWWLGEPAEDILQFLVLHLASLQLGLLL

Cc_imitator MPHSSLHPSIPHPRGHGARKAALVLLSVCLVTLWWLGEPPEDILQFLVLHLASLQLGLLL

Cjacchus MPHSSLHPSIPHPRGHGAQEAALVLLSVCLVTLWWLREAPEDILRFLVLHLASLQLGLLL

TitiM MPYSSLHPSIPRPRGHRAQEAALVLLSVCLVTLWWLGEPPEDILRFLVLHLASLQLGLLL

HowlerM MPYSSLHPSIPHPRGRRAQEAALVLLTVCLVTLWWLGEPPEDILRFLVLHLASLQLGLLL

*. ******** **: :::***.** .** *** * *..*. *: **::******* **

Chimp NGVCSLAEELRHIHSRYWGSYWRTVRACLGCPLRRGALLLLSIYFYYSLPNAVGPPFTWM

Bonobo NGVCSLAEELRHIHSRYWGSYWRTVRACLGCPLRRGALLLLSIYFYYSLPNAVGPPFTWM

Human NGVCSLAEELRHIHSRYRGSYWRTVRACLGCPLRRGALLLLSIYFYYSLPNAVGPPFTWM

Gggorilla NGLCSLAEELRHIHSRYRGSYWRTVRACLGCPLRRGALLLLSIYFYYSLPNAVGPPFTWM

Borang NGDCSLAEELRHIHSRYRGSYWRTVRACLGCPLRRGALLLLSSYFYYSLPNAVSVPFTWM

P_abelii NGVCSLAEELRHIHSRYRGSYWRTVRACLGCPLRRGALLLLSSYFYYSLPNAVSVPFTWM

WHgib NGVCSLAEELRHIHSRYRGSYWRTVRACLGCPLRCGALLLLSIYFYYSLPNAVGLPFTWM

AgileGib NGVCSLAEELRHIHSRYRGSYWRTVRACLGCPLRCGALLLLSIYFYYSLPNAVGLPFTWM

Siamang NGVCSLAEELRHIHSRYRGSYWRTVRACLGCPLRCGALLLLSIYFYYSLPNAVGLPFTWM

WCgib NGVCSLAEELRHIHSRYRGSYWRTVRACLGCPLRCGAVLLLSIYFYHSLPNAVGLPFTWM

Mleucoph NGVCSLAEELRHIHSRYRDSYWRTVRACLGCPFHHGTLLLLSGYFYYSLPNAVGLPFTWM

C_atys NGVCSLAEELRHIHSRYRDSYWRTVRACLGCPFHHGTLLLLSGYFYYSLPNAVGLPFTWM

P_anubis NGVCSLAEELRHIHSRYRDSYWRTVRACLGCPFHHGTLLLLSGYFYYSLPNAVGLPFTWM

BlackMan NGVCSLAEELRHIHSRYRDSYWRTVRACLGCPFHHGTLLLLSGYFYYSLPNAVGLPFTWM

Mnem NGVCSLAEELRHIHSRYRDSYWRTVRACLGCPFHHGTLLLLSGYFYYSLPNAVGLPFTWM

Mfascic NGVCSLAEELRHIHSRYRDSYWRTVRACLGCPFHHGTLLLLSGYFYYSLPNAVGLPFTWM

Mmull NGVCSLAEELRHIHSRYRDSYWRTVRACLGCPFHHGTLLLLSGYFYYSLPNAVGLPFTWM

Talapoin NGVCSLAEELRHIHSRYRDSYWRTVRACLGCPFHHGTLLLLSSYFYYSLPNAVGLPFTWM

WolfG NGVCSLAEELRHIHSRYRDSYWRTVRACLGCSFHRGTLLLLSSYFYYSLPNAVGLPFTWM

Chlor_sab KGVCSLAEELRHIHSRYRDSYWRTVRACLGCPFHHGTLLLLSSYFYYSLPNAVGLPFTWM

Rbieti NGVCSLAEELRHIHSRYRDSYWRTVQACLGCPLRCGTLLLLSSYFYCSLPNAVGLPFTWM

Rroxell NGVCSLAEELRHIHSRYRDSYWRTVQACLGCPLRCGTLLLLSSYFYCSLPNAVGLPFTWM

LeafM NGVCSLAEELRHIHSRYRDSYWRTVQACLGCPFRRGTLLLLSSYFYCSLPNAVGLSFTWM

Colobus NGVCSLAEELRHIHSRYRDSYWRTVQACLGCPFRRGTLLLLSSYFYYFLSNAFSLPFTWM

SquirrelM NRLCSLAEELRHIRSRYQGSYWRAVRACLGCPIRLGAQLVLSSYFYCSLPNAVGRPFTWT

Sbboliv NRLCSLAEELRHIRSRYQGSYWRAVRACLGCPSRLGAQLVLSSYFYCSLPNAVGRPFTWT

Cc_imitator NRFCSLAEELRHVHSRYQGSYWRAVRACLGCPIRLGAWLLLSSYFYCFLPNAVGRPFTWT

Cjacchus NRLCSLAEELRHVHTRYQGSYWRAVRACLGCPIRLGAQLLLSIYFYCFLPN--GRPFTWM

TitiM NRLCSLAEELRHVHSRYQGSYWRAVRACLGCPIRLGALLLLSSYFYCFRPNAVGWPFIWT

HowlerM NRLCSLAEELRHIRSRYQGSYWRAVRACLGCPIRLGAVLLLSSYFYCFLPTEVGRPFTWM

: *********:::** .****:*:*****. : *: *:** *** .. . .* *

Chimp LALLGLSQALNILLGLKGLAPAEISAVCEKGNFNVAHGLAWSYYIGYLRLILPELQARIR

Bonobo LALLGLSQALNILLGLKGLAPAEISAVCEKGNFNVAHGLAWSYYIGYLRLILPELQARIR

Human LALLGLSQALNILLGLKGLAPAEISAVCEKGNFNVAHGLAWSYYIGYLRLILPELQARIR

Gggorilla LALLGLSQALNILLGLKGLAPAEISAVCEKGNFNVAHGLAWSYYIGYLRLILPELQARIR

Borang LALLGLSQALNILLGLKGLAPAEISAVCEKGNFNVAHGLAWSYYIGYLRLILPGIQARIR

P_abelii LALLGLSQALNILLGLKGLAPAEISAVCEKGNFNVAHGLAWSYYIGYLRLILPGIQARIR

WHgib LALLGLSQALNILLGLKGLTPAEISAVCEKGNFNVAHGLAWSYYIGYLRLILPGLQARIR

AgileGib LALLGLSQALNILLGLKGLTPAEISAVCEKGNFNVAHGLAWSYYIGYLRLILPGLQARIR

Siamang LALLGLSQALNILLGLKGLTPAEISAVCEKGNFNVAHGLAWSYYIGYLRLILPGLQARIR

WCgib LALLGLSQALNILLGLKGLTPAEISAVCEKGNFNVAHGLAWSYYIGYLRLILPGLQARIR

Mleucoph LALLGLSQALNILFGLKGLTPAEISAVCEKGNFNVAHGLAWSYYIGYLRLILPGLQARIQ

C_atys LALLGLSQALNILFGLKGLTPAEISAVCEKGNFNVAHGLAWSYYIGYLRLILPGLQARIQ

P_anubis LALLGLSQALNILLGLKGLTPAEISAVCEKGNFNVAHGLAWSYYIGYLRLILPGLQARIQ

BlackMan LALLGLSQALNILLGLKGLTPAEISAVCEKGNFNVAHGLAWSYYIGYLRLILPGLQARIQ

Mnem LALLGLSQALNILLGLKGLTPAEISAVCEKGNFNVAHGLAWSYYIGYLRLILPGLQARIQ

Mfascic LALLGLSQALNILLGLKGLTPAEISAVCEKGNFNVAHGLAWSYYIGYLRLILPGLQARIQ

Mmull LALLGLSQALNILLGLKGLTPAEISAVCEKGNFNVAHGLAWSYYIGYLRLILPGLQARIQ

Talapoin LALLGLSQALNILLGLKGLTPAEISAVCEKGNFNVAHGLAWSYYIGYLRLILPGLQARIQ

WolfG LALLGLSQALNILLGLKGLTPAEISAVCEKGNFNVAHGLAWSYYIGYLRLILPGLQARIQ

Chlor_sab LALLGLSQALNILLGLKGLTPAEISAVCEKGNFNVAHGLAWSYYIGYLRLILPGLQARIQ

Rbieti LALLGLAQAVNILLGLKGLTPAEISAVCEKGNFNVAHGLAWSYYIGYLQLILPGLQARIQ

Rroxell LALLGLGQALNILLGLKGLTPAEISAVCEKGNFNVAHGLAWSYYIGYLQLILPGLQARIQ

LeafM LALLGLSQALNILLGLKGLTPAEISAVCEKGNFNVAHGLAWSYYIGYLQLILPGLQARIQ

Colobus LALLGLSQALNILLGLKGLTPAEISAVCEKGNFNVAHGLAWSYYIGYLQLILPGLQARIQ

SquirrelM LALLGFSQALNILLDLKGLAPAEISAVCEKGNFNVAHGLAWSYYIGYLRLILPGFQARIR

Sbboliv LALLGFSQALNILLDLKGLAPAEISAVCEKGNFNVAHGLAWSYYIGYLRLILPGFQARIR

Cc_imitator LALLGFSQALNVLLGLKGLAPAEISAVCEKGNFNVAHGLAWSYYIGYLRLILPGFQARIR

Cjacchus LALLGFSQALNILLGLKGLAPAEISAVCEKRNFNVAHGLAWSYYIGYLRLILPGFQARIR

TitiM LALLGLSQALNILLDLKGLAPAEISAVCEKGNFNVAHGLAWSYYIGYLRLILPGFQARIR

HowlerM LALLGLSQALNILLGLKGLAPAEISAVCEKGNFNVAHGLAWSYYIGYLRLILPGFQARIR

*****:.**:*:*:.****:********** *****************:**** :****:

Chimp TYNQHYNNLLRGAVSQRLYILLPLDCGVPDNLSMADPNIRFLDKLPQQTADRAGIKDRVY

Bonobo TYNQHYNNLLRGAVSQRLYILLPLDCGVPDNLSMADPNIRFLDKLPQQTADRAGIKDRVY

Human TYNQHYNNLLRGAVSQRLYILLPLDCGVPDNLSMADPNIRFLDKLPQQTGDHAGIKDRVY

Gggorilla TYNQHYNNLLRGAVSQRLYILLPLDCGVPDNLSMADPNIRFLDKLPQQTADRAGIKDRVY

Borang TYNQHYNNLLWGAVSQRLYILLPLDCGVPDNLSMADPNIRFLDKLPQQTADRAGIKDRVY

P_abelii TYNQHYNNLLWGAVSQRLYILLPLDCGVPDNLSMADPNIRFLDKLPQQTADRAGIKDRVY

WHgib TYNQHYNNLLRGAVSQRLYILLPLDCGVPDNLSMADPNIRFLDKLPQQTADRAGIKDRVY

AgileGib TYNQHYNNLLRGAVSQRLYILLPLDCGVPDNLSMADPNIRFLDKLPQQTADRAGIKDRVY

Siamang TYNQHYNNLLRGTVSQRLYILLPLDCGVPDNLSMADPNIRFLDKLPQQTADRAGIKDRVY

WCgib TYNQHYNNLLRGAVSQRLYILLPLDCGVPDNLSMADPNIRFLDKLPQQTADRAGIKDRVY

Mleucoph TYNQHYNNLLRGAVSQRLYILLPLDCGVPDNLSMADPNIRFLDKLPQQTADRAGVKDRVY

C_atys TYNQHYNNLLRGTVSQRLYILLPLDCGVPDNLSMADPNIRFLDKLPQQTADRAGIKDRVY

P_anubis TYNQHYNNLLRGAVSQRLYILLPLDCGVPDNLSMADPNIRFLDKLPQQTADRAGIKDRVY

BlackMan TYNQHYNNLLRGAVSQRLYILLPLDCGVPDNLSMADPNIRFLDKLPQQTADRAGIKDRVY

Mnem TYNQHYNNLLRGAVSQRLYILLPLDCGVPDNLSMADPNIRFLDKLPQQTADRAGIKDRVY

Mfascic TYNQHYNNLLRGAVSQRLYILLPLDCGVPDNLSMADPNIRFLDKLPQQTADRAGIKDRVY

Mmull TYNQHYNNLLRGAVSQRLYILLPLDCGVPDNLSMADPNIRFLDKLPQQTADRAGIKDRVY

Talapoin TYNQHYNNLLRGAVSQRLYILLPLDCGVPDNLSMADPNIRFLDKLPQQTTDRAGIKDRVY

WolfG TYNQHYNNLLRGAVSQRLYILLPLDCGVPDNLSMADPNIRFLDKLPQQTTDRAGIKDRVY

Chlor_sab TYNQHYNHLLRGAVSQRLYILLPLDCGVPDNLSMADPNIRFLDKLPQQTADRAGIKDRVY

Rbieti TYNQRYNNLLRGAVSQRLYILLPLDCGVPDNLSMADPNIRFLDKLPQQTADRAGIKDRVY

Rroxell TYNQRYNNLLRGAVSQRLYILLPLDCGVPDNLSMADPNIRFLDKLPQQTADRAGIKDRVY

LeafM TYNQRYNNLLRGAVSQRLYILLPLDCGVPDNLSMADPNIRFLDKLPQQTADRAGIKDRVY

Colobus TYNQHYNNLLRGAVSQRLYILLPLDCGVPDNLSMADPNIRFLDKLPQQTADRAGIKDRVY

SquirrelM TYNQHNNNVLRGPASQRLYILFPLDCGVPDNLSTADPNIRFLGKLPQQTIDRAGIKERVY

Sbboliv TYNQHNNNVLRGPASQRLYILFPLDCGVPDNLSAADPNIRFLGKLPQQTIDRAGIKERVY

Cc_imitator TYNQHNNNVLRGPASQRLYILFPLDCGVPDNLSTADANIRFLDKLPQQTIDRAGIKGRVY

Cjacchus TYNQHNNNVLRGPASQRLYILFPLDCGVPDNLSTADPNIRFLDKLPQETIDRAGIKGRVY

TitiM TYNQQNNNVLRGPASQRLYILFPLDCGVPDNLSTADPNIRFLDKLPQQTIDRAGIKGRVY

HowlerM TYNQHNNNVLRGPASQRLYILFPLDCGVPDNLSTADPNIRFLDKLPQQTIDRAGIKGRVY

****: *::* *..*******:*********** **.*****.****:* *:**:* ***

Chimp SNSIYELLENGQRAGTCVLEYATPLQTLFAMSQYSQAGFSREDRLEQAKLFCRTLEDILA

Bonobo SNSIYELLENGQRAGTCVLEYATPLQTLFAMSQYSQAGFSREDRLEQAKLFCRTLEDILA

Human SNSIYELLENGQRAGTCVLEYATPLQTLFAMSQYSQAGFSREDRLEQAKLFCRTLEDILA

Gggorilla SNSIYELLENGQRAGTCVLEYATPLQTLFAMSQCSQAGFSREDRLEQAKLFCRTLEDILA

Borang SNSIYELLENGQRAGTCVLEYATPLQTLFAMSQYGQAGFSREDRLEQAKLFCRTLEDILA

P_abelii SNSIYELLENGQRAGTCVLEYATPLQTLFAMSQYGQAGFSREDRLEQAKLFCRTLEDILA

WHgib SNSIYELLENGQRAGTCVLEYATPLQTLFAMSQHGQAGFSREDRLEQAKLFCRTLEDILA

AgileGib SNSIYELLENGQRAGTCVLEYATPLQTLFAMSQHGQAGFSREDRLEQAKLFCRTLEDILA

Siamang SNSIYELLENGQRAGTCVLEYATPLQTLFAMSQHGQAGFSREDRLEQAKLFCRTLEDILA

WCgib SNSIYELLENGQRAGTCVLEYATPLQTLFAMSQHGQAGFSREDRLEQAKLFCRTLEDILA

Mleucoph SNSIYELLENGQRAGTCVLEYATPLQTLFAMSQYGQAGFSREDRLEQAKLFCRTLEDILA

C_atys SNSIYELLENGQRAGTCVLEYATPLQTLFAMSQYGQAGFSREDRLEQAKLFCRTLEDILA

P_anubis SNSIYELLENGQRAGTCVLEYATPLQTLFAMSQYGQAGFSREDRLEQAKLFCRTLEDILA

BlackMan SNSIYELLENGQWAGTCVLEYATPLQTLFAMSQYGQAGFSREDRLEQAKLFCRTLEDILA

Mnem SNSIYELLENGQWAGTCVLEYATPLQTLFAMSQYGQAGFSREDRLEQAKLFCRTLEDILA

Mfascic SNSIYELLENGQRAGTCVLEYATPLQTLFAMSQYGQAGFSREDRLEQVKLFCRTLEDILA

Mmull SNSIYELLENGQRAGTCVLEYATPLQTLFAMSQYGQAGFSREDRLEQVKLFCRTLEDILA

Talapoin SNSIYELLENGQRAGTCVLEYATPLQTLFAMSQYGQAGFSREDRLEQAKLFCRTLEDILA

WolfG SNSIYELLENGQRAGTCVLEYATPLQTLFAMSQYGQAGFSREDRLEQAKLFCRTLEDILA

Chlor_sab SNSIYELLENGQRAGTCVLEYATPLQTLFAMSQYGQAGFSREDRLEQAKLFCRTLEDILA

Rbieti SNSIYELLENGQRAGTCVLEYATPLQTLFAMSQHGQAGFSREDRLEQAKLFCRTLEDILA

Rroxell SNSIYELLENGQRAGTCVLEYATPLQTLFAMSQHGQAGFSREDRLEQAKLFCRTLEDILA

LeafM SNSIYELLENGQRAGTCVLEYATPLQTLFAMSQHGQAGFSREDRLEQAKLFCRALEDILA

Colobus SNSIYELLENGQRAGTCVLEYATPLQTLFAMSQHGQAGFSREDRLEQAKLFCRTLEDILA

SquirrelM TNSIYELLENGQRAGACVLEYASPLQTLFAMSQYGQAGFSREDRLEQAKLFCRTLEDILA

Sbboliv TNSIYELLENGQRAGACVLEYASPLQTLFAMSQYGQAGFSREDRLEQAKLFCRTLEDILA

Cc_imitator TNSIYELLENGQRAGACVLEYASPLQTLFAMSQYGQAGFSREDRLEQAKLFCRTLEDILA

Cjacchus TNSIYELLENGQRAGACVLEYASPLQTLFAMSQYGQAGFSREDRLEQAKLFCRTLEDILA

TitiM TNSIYELLENGQRAGACVLEYASPLQTLFAMSQYGQAGFSRKDRLEQAKLFCRTLEDILA

HowlerM TNSIYELLENGQRAGACVLEYASPLQTLFAMSQYGQAGFSREDRLEQAKLFCRTLEDILA

:*********** **:******:********** .******:*****.*****:******

Chimp DAPESQNNCRLIAYQEPADDSSFSLSQEVLRHLRQEEKEEVTVGSLKTSAVPSTSTMSQE

Bonobo DAPESQNNCRLIAYQEPADDSSFSLSQEVLRHLRQEEKEEVTVGSLKTSAVPSTSTMSQE

Human DAPESQNNCRLIAYQEPADDSSFSLSQEVLRHLRQEEKEEVTVGSLKTSAVPSTSTMSQE

Gggorilla DAPESQNNCRLIAYQEPADDSSFSLSQEVLRHLRQEEKEEVTVGSLKTSAVPSTSTMSQE

Borang DAPECRNNCRLIAYQEPADDSSFSLSQEVLRHLRQEEKEEVTVGSLKTSAVPSTSMMSQE

P_abelii DAPECRNNCRLIAYQEPADDSSFSLSQEVLRHLRQEEKEEVTVGSLKTSAVPSTSMMSQE

WHgib DAPESQNNCRLIAYQEPADDSSFSLSQEVLRHLRQEEKEEVTVGSLKTSAVPSTSTMSQE

AgileGib DAPESQNNCRLIAYQEPADDSSFSLSQEVLRHLRQEEKEEVTVGSLKTSAVPSTSTMSQE

Siamang DAPESQNNCRLIAYQEPADDSSFSLSQEVLRHLRQEEKEEVTVGSLKTSAVPSTSTMSQE

WCgib DAPESQNNCRLIAYQEPADDSSFSLSQEVLRHLRQEEKEEVTVGSLKTSAVPSTSTMSQE

Mleucoph DNPESQNNCRLIVYPEPADDSSFSLSQEVLRHLRQEEKEEVTVGNLKNSAVPSTSTMSQE

C_atys DNPESQNNCRLIVYPEPADDSSFSLSQEVLRHLRQEEKEEVTVGSLKNSAVPSTSTMSQE

P_anubis DNPESQNNCRLIVYPEPADDSSFSLSQEVLRHLRQEEKEEVTVGSLKNSAVPSTSTMSQE

BlackMan DNPESQNNCRLIVYQEPADDSSFSLSQEVLRHLRQEEKEEVTVGSLKNSAVPSTSTMSQE

Mnem DNPESQNKCRLIVYLEPADDSSFSLSQEVLRHLRQEEKEEVTVGSLKNSAVPSTSTMSQE

Mfascic DNPESQNNCRLIVYSEPADDSSFSLSQEVLRHLRQEKKEEVTVGSLKNSAVPSTSTMSQE

Mmull DNPESQNNCRLIVYSEPADDSSFSLSQEVLRHLRQEEKEEVTVGSLKNSAVPSTSTMSQE

Talapoin DNPESQNNCRLIVYLEPADDSSFSLSQEVLRHLRQEEKEEVTVGSLKNSAVPSTSTMSQE

WolfG DNPESQNNCRLIVYLEPADDSSFSLSQEVLRHLRQEEKEEVTVGSLKNSAVPSTSTMSQE

Chlor_sab DNPESQNNCRLIVYLEPADDSSFSLSQEVLQHLRQEEKEEVTVGSLKNSAVPSTSTMSQE

Rbieti DNPESQNNCRLIVYQEPADDSSFSLSQEVLRHLRQEEKEEVTVGSLKTSGAPSTSTMSQE

Rroxell DNPESQNNCRLIVYQEPADDSSFSLSQEVLRHLRQEEKEEVTVGSLKTSGAPSTSTMSQE

LeafM DNPESQNNCRLIVYQEPADDSSFSLSQEVLRHLRQEEKEEVTVGSLKTSAAPSTSTMSQE

Colobus DNPESQNNCRLIVYQEPADDSSFSLSQEVLRHLRQEEKEEVTVGSLKTSAVPSTSTMSQE

SquirrelM DAPESQNNCRLIVYQEPADGSSFLLSQEVLRHLRQEEEEEVTVGSLKTTEVPSTSTMSQE

Sbboliv DAPESQNNCRLIVYQEPADGSSFLLSQEVLRHLRQEEEEEVTVGSLKTTEVPSTSTMSQE

Cc_imitator DAPESQNNCRLIVYQEPADGSSFLLSQEVLRHLRQEEEEEVTVGSLKTSEVPISSTMSQE

Cjacchus DAPESQNNCRLIVYEEPADGSSFLLSQEVLQHLRQEEEEEVTVGSLKTSEVPSTSTMSQE

TitiM DAPESQNNCRLIVYQEPADGSSFLLSQEVLRHLRQEEEEEVTVGSLKTSEVPSTSTMSQE

HowlerM DAPESQNNCRLIVYQEPADGSSFLLSQEVLRHLRQEEEEEVTVGSFKTSQAPSTSTMSQE

* **.:*:****.* ****.*** ******:*****::******.:*.: .* :* ****

Chimp PELLISGMEKPLPLRTDFS

Bonobo PELLISGMEKPLPLRTDFS

Human PELLISGMEKPLPLRTDFS

Gggorilla PELLISGMEKPLPLRTDFS

Borang PELLISGMEKPLPLHTDFS

P_abelii PELLISGMEKPLPLHTDFS

WHgib PELLISGLEKPLPLRTDFS

AgileGib PELLISGLEKPLPLRTDFS

Siamang PELLISGLEKPLPLRTDFS

WCgib PELLISGLEKPLPLRTDFS

Mleucoph PELLISGMEKPLPLRTDFS

C_atys PELLISGMEKPLPLRTDFS

P_anubis PELLISGMEKPLPLRTDFS

BlackMan PELLISGMEKPLPLRTDFS

Mnem PELLISGMEKPLPLRTDFS

Mfascic PELLISGMEKPLPLRTDFS

Mmull PELLISGMEKPLPLRTDFS

Talapoin PELLISGMEKPLPLRTDFS

WolfG PELLISGMEKPLPLRTDFS

Chlor_sab PELLISGMEKPLPLRTDFS

Rbieti PELLISGMEKPLPLRTDFS

Rroxell PELLISGMEKPLPLRTDFS

LeafM PELLISGMEKPLPLRTDFS

Colobus PELLISGMEKPLPLRTDFS

SquirrelM PELLISGMEKPLPLRSDLF

Sbboliv PELLISGMEKPLPLRSDLF

Cc_imitator PELLISGMEKPLPLRSDLF

Cjacchus PELLISGMEKPLPLRSDLF

TitiM LELLISGMEKPLPLRSDLS

HowlerM PELLISGMEKPLPLRSDLS

******:******::*:
